# Increasing Clinical Trial Participation of Black Women Diagnosed with Breast Cancer

**DOI:** 10.1007/s40615-023-01644-z

**Published:** 2023-06-14

**Authors:** Ricki Fairley, James W. Lillard, Alexandra Berk, Sophia Cornew, Joseph Gaspero, James Gillespie, LaTrisha L. Horne, Sabrina Kidane, Sandra B. Munro, Matthew Parsons, Emily R. Powers, Suzanne E. Rizzo, Alyson Tishcler, Hope Wohl, Marisa C. Weiss

**Affiliations:** 1 TOUCH, The Black Breast Cancer Alliance, Annapolis, MD USA; 2 Department of Microbiology, Biochemistry, and Immunology, Morehouse School of Medicine, Atlanta, GA USA; 3 Medical Affairs, Invitae, San Francisco, CA USA; 4 Patient Network and Data, Invitae, San Francisco, CA USA; 5 Center for Healthcare Innovation, Chicago, IL USA; 6 LRW A Material Company, Los Angeles, CA USA; 7 Breastcancer.org, Ardmore, PA USA; 8 Lankenau Medical Center, Wynnewood, PA USA

**Keywords:** Breast Cancer, Black/African American Women, Clinical Trials, Health Disparities

## Abstract

Despite racial disparities in breast cancer mortality, Black women remain underrepresented in clinical trials. In this mixed methods research, 48 Black women were engaged via focus group discussions and in-depth interviews to better understand the lived experience of women with breast cancer. The results of this qualitative study informed the development of a subsequent online survey to identify barriers, motivators, and other factors that influence decision-making by Black women diagnosed with breast cancer when considering clinical trial participation. Among the 257 Black survey participants, most (95%) were aware of clinical trials; of those, most viewed them as lifesaving (81%) and/or benefiting others (90%). Negative perceptions such as serious side effects (58%), not receiving real treatment (52%), or risk of potential harm (62%) were indicated. Barriers included financial expenses (49%), concerns that their condition could be made worse (29%), that they would receive a placebo (28%), or that treatment was unapproved (28%). Participants were more likely than their health care providers (HCPs) to initiate discussions of clinical trials (53% versus 33%), and 29% of participants indicated a need for more information about risks and benefits, even after having those conversations. The most trustworthy sources of information on clinical trials were HCPs (66%) and breast cancer support groups (64%). These results suggest that trusted communities are key for providing education on clinical trials. However, there is also a need for HCPs to proactively discuss clinical trials with patients to ensure that they are adequately informed about all aspects of participation.

**Supplementary Information:**

The online version contains supplementary material available at 10.1007/s40615-023-01644-z.

## Introduction

Black women in the United States are 42% more likely to die from breast cancer than non-Hispanic White women [1, 2]. Disparities in health outcomes between Black and White Americans have long existed due to structural racism and discrimination, which have led to socioeconomic disadvantages and lack of access to health care [3]. Yet even when access-related factors such as income and health insurance are controlled for, health outcomes among Black Americans remain worse than those for White Americans [4].

In addition to socioeconomic factors, biological differences have been linked to increased breast cancer mortality in Black women. Black women are more likely to be diagnosed with triple-negative breast cancer, a more aggressive type of breast cancer that has fewer treatment options and relatively poor prognosis when compared with other types of breast cancer [5, 6]. Germline pathogenic variants in the *BRCA1* and *BRCA2* breast cancer predisposition genes are more common in Black women than in White women, except for women of Ashkenazi Jewish ancestry [7]. Yet Black women are less likely than White women to be offered genetic testing [8–10], even though results can serve as a foundation for screening, surveillance, prophylactic procedures, and, increasingly, innovative targeted therapies [11].

Screening and disease management can also contribute to racial disparities in breast cancer mortality. Black women are more likely than White women to be diagnosed with breast cancer before the age of 50 [12]. However, it wasn’t until 1997 that the American Cancer Society (ACS) changed its decades-old recommendation that women only begin annual mammograms when they reach 50 years of age [13]. Presently, annual mammograms are recommended for women aged 45-54 years and considered optional for those aged 40-44 years [13]. In addition, significant racial differences in the receipt of appropriate cancer treatments have been reported [4, 14].

Clinical trials are essential for evaluating the safety and efficacy of new cancer treatments [15]. They also provide patients with opportunities to access the newest innovations in treatments, including precision therapies that may be uniquely targeted to their disease [16]. Racial minority groups have long been underrepresented in cancer research trials [17, 18]. A systematic review of participation in clinical trials found that Black patients comprised less than 4% of all patients enrolled in clinical trials for emerging immune-based cancer treatments [19, 20]. Factors contributing to low enrollment of underrepresented populations occur at health care system, physician, and patient levels [21–23]. Key barriers include insufficient access to care [24]; financial constraints [21]; lack of knowledge regarding trial availability [21, 25]; lack of transportation and childcare [26, 27]; lack of diversity of health care providers [28]; and provider time constraints, attitudes, and implicit bias [29, 30]. Apprehension based on cultural differences, perceptions of discrimination, or medical mistrust have also been attributed to patients’ decisions not to participate in clinical trials [31–34]. Yet in research conducted by a National Cancer Institute (NCI)-designated cancer center in collaboration with community partnerships, most Black individuals were likely to agree to participate in biomedical research when asked [35, 36].

Persistent lack of enrollment of underrepresented populations in clinical trials has led clinical trial consortia and cancer research organizations to prioritize equal access to trials [37, 38]. The National Institutes of Health (NIH) Revitalization Act of 1993 mandated the inclusion of women and other underrepresented populations in all NIH-sponsored clinical trials (“[39]). Subsequent efforts have included increasing financial assistance, improving access to clinical services, addressing cultural barriers, and improving physician outreach [40]. However, analysis of enrollment in clinical trials has indicated little improvement, as a study performed a decade later demonstrated that only 2% of approximately 10,000 NCI trials had a sufficient proportion of minority participants to meet the NIH’s mandated goals [41]. A 2022 consensus study report by the National Academy of Sciences and the NIH found that, despite the priority of increasing diversity in clinical trials, the majority of participants continue to be White men [42].

Despite previous studies that have identified barriers and perceptions leading to lack of participation in clinical trials by Black individuals, there remains a paucity of information on why Black women in particular choose not to participate in clinical research [43]. It is important to continue to find innovative engagement strategies to determine and incorporate participants’ perceptions and needs. This mixed method research represents a collaboration among breast cancer patient advocacy organizations, historically Black colleges and universities (HBCUs), non-profits, and industry collaborators to address this issue. Our key objective was to engage Black women to examine clinical trial awareness and perceptions; to identify various cultural, emotional, and social barriers and motivators to participation; and to identify and quantify levels of mistrust in medical research. In addition, we sought to identify actionable insights, using patients’ voices, for outreach, messaging, and engagement.

## Methods

This mixed methods research—composed of a qualitative study, a subsequent quantitative survey study, and patient electronic data analysis for a subset of survey participants—was conducted nationwide by a marketing research firm (Material Holdings, LLC, Los Angeles, CA) and approved by an independent institutional review board (Pearl IRB, Indianapolis, IN). Participants were recruited through social media, online survey panels, Ciitizen, or patient advocacy groups including TOUCH, The Black Breast Cancer Alliance, and Breastcancer.org. Respondents who were employed by a marketing or market research company; a company that makes or distributes pharmaceutical products; or a hospital, medical clinic, doctor's office, or any other job in the medical field were excluded. Participants provided their written consent after being informed about all study procedures and data privacy guidelines.

### Qualitative Study to Inform Survey Questions

An exploratory, online qualitative study was conducted from April 12 to April 22, 2021, to understand the lived experience of Black women with breast cancer.

Women who self-identified as African American or Black, were aged 25 to 70 years, and had never participated in a clinical trial were eligible to participate. Participants also had to have been treated for breast cancer within the past 5 years, been informed by a doctor that they were at risk of breast cancer, or had at least one close female family member diagnosed with breast cancer. The study consisted of 14 online focus group discussions with up to six participants each, plus six individual, semi-structured in-depth interviews. The focus group discussions and in-depth interviews were designed to last 120 and 60 min, respectively (Supplemental Methods), and were conducted by a known leader in the Black breast cancer patient advocacy space, to facilitate trust and openness. All discussions and interviews were observed live and recorded. Session transcripts were systematically reviewed, and attitudes and behaviors from each topic area were coded to identify common themes around perceptions of clinical trials, levels of knowledge about breast cancer and related statistics and treatment options, and the impact of specific messaging on respondents’ interest in and consideration of clinical trial participation. Salient themes that emerged from the qualitative study were incorporated into the design of the subsequent quantitative survey study.

### Quantitative Survey Study

Based on findings from the qualitative study, a quantitative study consisting of a 25-min online survey was conducted from June 23, 2021, to August 9, 2021. Participants were women or men who self-identified as African American or Black, were at least 18 years old, and had been diagnosed with breast cancer. Participants were currently in treatment for or in remission from any stage of breast cancer. The survey addressed the following topics: clinical trial awareness, perceptions, and expectations; barriers and motivators to participation in clinical trials; relationships with health care providers (HCPs); discussions about clinical trials with HCPs; and community engagement and resources (Supplemental Methods). Statistically significant differences in clinical trial participation based on stage of cancer and income, as well as differences in levels of trust in various sources of information were determined by t-test analysis, indicated by *p* < 0.05.

### Ciitizen Patient Electronic Data Analysis

A subset of survey participants were enrolled in Ciitizen (now part of Invitae, San Francisco, CA). Ciitizen leverages the HIPAA right of access on behalf of patients to collect and store their medical records, and turns medical record documents into structured, longitudinal data that can be shared with whomever the patients want, for their own clinical treatment or for observational research and clinical trials. When a patient signs up for Ciitizen, they provide the name of their sites of care and complete a HIPAA-compliant request form authorizing Ciitizen to request medical records on their behalf. These records are then uploaded to the patient’s Ciitizen account for their own use. As part of the Ciitizen offering, key clinical and treatment data are extracted from the patient’s medical records to create a Ciitizen summary, which provides the patient with a visually friendly summary of their treatment journey that can be used for second opinions or other personal care coordination needs. The primary purpose of Ciitizen’s data extraction into the Ciitizen summary format is for the patient’s personal use. A possible secondary use of the extracted data is for research purposes, for patients who have consented to share their data for research use.

In addition to receiving their own medical records, survey participants who signed up for Ciitizen consented to share their de-identified data for the study. Clinical data from Ciitizen was used to describe the study population’s clinical features, including molecular type of cancer, medications, therapeutic procedures, comorbidities, and adverse events. If a clinical feature was reported at least once in the patient’s Ciitizen summary, it was included in calculating percentages for that feature. Of note, the absence of variables from the Ciitizen dataset does not mean they were not a part of the participant’s clinical history.

## Results

### Qualitative Study

Forty-eight women participated in the qualitative study. Most were aged < 55 years (85%) and were diagnosed with stage II/III or stage IV breast cancer (44%) (Table 1). The themes that emerged centered on perceptions of the healthcare system, knowledge of clinical trials, and barriers to and motivators of clinical trial participation.

**Table 1:** Participant demographics

	Qualitative study (*n*=48)	Quantitative study (*n*=257)
Sex assigned at birth—no.		
Female	48	256
Male	0	1
Age—yr (%)		
25-34	15 (31)	21(8)
35-44	13 (27)	92 (36)
45-54	13 (27)	83 (32)
55-64	4 (8)	40 (16)
65-70	3 (6)	13 (5)
71-75	0	5 (2)
≥ 76	0	3 (1)
Breast cancer stage (at time of survey)—no. (%)		
Stage I	0	29 (11)
Stage II/III	11 (23)	53 (21)
Stage IV	10 (21)	117 (46)
In remission of Stage I-III	8 (17)	55 (21)
In remission of Stage IV	0	3 (1)
Family member of Stage II/III breast cancer patient	6 (13)	-
Family member of Stage IV breast cancer patient	4 (8)	-
At risk for breast cancer	9 (19)	-
Self-reported race/ethnicity^a^ —no. (%)		
Black/African American	48 (100)	257 (100)
White	0	10 (4)
Native American or Alaskan	0	5 (2)
Combined annual household income^b^—no. (%)		
< $25K	5 (17)	-
$25–49K	6 (21)	-
< $35K	-	44 (17)
$35–49K	-	14 (5)
$50–74K	9 (31)	47 (18)
≥ $75K	9 (31)	147 (58)
Did not answer	0	5 (2)
Employment status^c^—no. (%)		
Full-time	-	164 (64)
Part-time	-	13 (5)
Not employed	-	18 (7)
Retired	-	28 (11)
On disability	-	28 (11)
Homemaker	-	3 (1)
Currently seeking work		3 (1)
Education^b^—no. (%)		
High school	0	21 (8)
Vocational/trade school	0	8 (3)
Some college	5 (17)	56 (22)
College graduate	13 (45)	90 (35)
Associate degree	5 (17)	0
Post-graduate degree	6 (21)	82 (32)
Health care insurance^b^—no. (%)		
Commercial through work or union	16 (55)	75 (29)
Affordable Care Act plan	0	23 (9)
Medicare only	1 (3)	82 (32)
Medicare HMO/Advantage	1 (3)	0
Medicaid	9 (31)	67 (26)
Veteran’s or active military healthcare	1 (3)	5 (2)
No health insurance	1 (3)	5 (2)

^a^ Participants could select all responses that applied

^b^ Answers were collected only from patient respondents (i.e., not family members or those at risk) for the qualitative study (*n*=29)

^c^ Employment status was not recorded in the qualitative study

#### Perceptions of the Healthcare System

Overall, participants perceived that the established institutional healthcare system did not serve them as well as other racial groups, which stemmed from historical experiences within the Black community and recent personal experiences.

Participants reported that there was a historical lack of support from the medical community and an ill-established rapport with Black women that resulted in skepticism of the established healthcare system. For example, one participant said,

“*[Black women] really don't trust the science and they see a lot of pharmaceutical companies making a lot of money and not coming down to the communities unless they are doing some kind of trial. They don't see you outside of a trial helping educate your community; just take the money. They don't like it. That's why you have a lot of people in breast cancer organization. It just feels like they've been talking about research all these years, they don't even come down and support or educate.”*

Further, historical traumas within the Black community were cited by participants, with references most commonly to Henrietta Lacks and the Tuskegee syphilis study. For example,

*“I think of the Tuskegee experiment, honestly, when I think of clinical trials, as much as it's I know not the same thing (…). I'm very, very weary of the medical field and trusting comes after time.”*

*“And for some reason my mind always goes back to the Tuskegee experiment. Like we're going to put this in you, see how it works, and then there could be some kind of negative effect or negative results from it. Tuskegee always pops up in my head anyway.”*

Negative personal experiences that had impacted participants’ perceptions of the healthcare system included a lack of trust in medical teams and a lack of tailored treatment approaches. For example,

*“I always go for, how do they make me feel, what's their bedside manner? Do I feel like you actually care about me as a person, and I'm not just another name on your tablet or a piece of paper with a diagnosis?”*

Although racial preference for medical care teams varied, several participants noted that they trust non-White more than White HCPs to provide individualized care. For example, one participant stated,

*“I realize that I do prefer a person of color. And honestly, I don't think I even care if it's Black or Asian or whatever, it doesn't really matter. I've found that even the doctor who just recently did my hysterectomy was from Asian descendants. She was just very, she made sure and she pointed out something very plainly that was different because I was a person of color that she was going to do differently in the surgery. And that, to me, it was like, you addressed it. And you saw me as a person and you saw my color and was able to give me better care and more personalized care, if that makes sense.”*

Related to these perceptions, participants did not believe they had enough information about their diagnoses and treatment options. This perception appeared to be based on feeling overwhelmed upon initial diagnosis, which made it difficult for participants to retain information and subsequently left them feeling that their medical team didn’t share enough details. For example, one participant recalled having difficulty remembering details about her relative’s diagnosis and treatments,

*“She’s stage…What did I say? Three is the…I’m not into all the…I just go and be supportive and help her out…And I’m not sure if it’s stage…It’s stage four. Yeah. It’s stage four. But when you asked the other question, I said three?”*

Further, participants were generally unaware that mortality rates are higher among Black women than White women. For example, two participants noted,

*“I think cancer doesn’t discriminate between you if you're White, Black, Asian, Hispanic. It just doesn’t discriminate (...). And maybe I shouldn’t say it this way, but we’re all going to pass away.”*

*“In my recent visit in the cancer center, and granted, there were different forms of cancer there that were being treated. But I just saw people from all walks of life.”*

#### Knowledge of Clinical Trials

Participants indicated that the topic of clinical trials was left out of discussions with HCPs regarding treatment options. For example, one participant noted,

*“Whenever I would hear ‘clinical trial,’ I would always think ‘experiment’ because it was never really broken down to me, I never considered it, and I’ve never been approached personally to participate.”*

In addition, misconceptions about clinical trials emerged among the participants, seemingly because of misinformation circulating through family, personal communities, the breast cancer community, and personal medical care teams. Importantly, these misconceptions led to hesitation to participate in clinical trials.

As an example, some participants did not know that research and evidence are required before a treatment can be approved for clinical trials,

*“If it would have been shared we’ve been doing it for this many years and this is the data we have, I would have been more open.”*

Further, participants noted that their communities share the false notion that participation in a clinical trial means becoming a “guinea pig” in an experiment, and that seeing the data would help overcome this misconception. For example, two participants noted,

*“I need, like, data and information and statistics and things like that. Yeah, because I, I mean, obviously most people I’m sure want to find a cure for this, but a lot of people would be hesitant to put themselves out there as the sort of Guinea pig.”*

*“Feels like you got to pack up and move somewhere and they watch you through a glass…too many movies. Everything is white and sterile.”*

Another common misconception was that clinical trial participants can be randomly assigned to receive no treatment (i.e., placebo or sugar pills). Relatedly, participants were unfamiliar with the term “standard of care” and that it is guaranteed in trials they would participate in. For example,

*“Clinical trial we know is a trial and some people get the A, B, and C drug and some people get the A, B, and sugar drug. So I think that’s our biggest fear with doing all of this and then I’m not getting the real deal (...). Getting the placebo or whatever it is (...). I mean, the only thing is, and I understand how Black people don’t want to do trials, is because I know that to prepare for a trial, it’s a lot of work and it’s very regimented that you have to do all these things. And then just to find out at the end that you didn’t get the actual drug. You know, that’s pretty discouraging.”*

In addition, participants noted strong religious beliefs, which led to an emphasis on faith and prayer over experimental science. As a result, the idea of a clinical trial can be rejected as an option before it can be fully considered. For example, two participants who had family members with breast cancer noted,

*“I believe in God, but they think that if you pray everything away, it's going to go away. If the doctor doesn't. And it's a backwards thing to me, it's like, if the doctor doesn't say it, or the doctor don't fix it, then we going to pray to God. But no holistic treatment, clinical trials, nothing else. It has to be either from the doctor or from the Lord. So clinical trials, they just... I don't know, it seems like they demonize it in a way. They're not open-minded."*

*“When she was first diagnosed, it was like I don't have long to live. According to the doctors and we said, no, we're not going by the doctor. We're going by what God says and we're going to pray. We started a prayer group and that's what we've been doing.”*

#### Barriers to and Motivators of Clinical Trial Participation

In response to educational messaging regarding clinical trials, five overarching barriers were identified. The first two barriers were denial and fear of breast cancer diagnoses, which led to a reluctance to learn more. Some participants noted that they wanted to continue living as normally as possible, leading to not sharing their diagnosis with others and in some cases not initially seeking treatment. Even high-risk participants who did not have a breast cancer diagnosis expressed fear of learning their status. The third barrier was composed of logistical challenges related to time (i.e., disruptions to daily life, career, and family), proximity, and self-imposed qualifications (i.e., delaying approved treatments while waiting to see if they are eligible for a trial). A fourth barrier centered on privacy, with both personal and cultural implications. Personally, participants noted that others would find out about their diagnoses if they took part in a clinical trial, and might think of them differently as a result. Participants also noted that the culture of privacy in the Black community resulted in participants not knowing their complete family medical history. The fifth barrier noted by participants was a lack of access to medical care, both for preventative care and for treatment, with references to access being driven by insurance coverage.

Messages that were most persuasive were those that conveyed clinical trials in a more relatable context, or evoked emotion to help patients think beyond themselves. For example, messages related to the theme “Everything has been a trial” provides a perhaps unconsidered perspective of clinical trials. One participant noted,

*“This is an idea of what the process looks like. It lets me know that I’m not the first person, or before it even gets to me, before it even gets to humans, it’s been tested for a minimum of 6 years, which is important. That makes me feel not like a guinea pig.”*

Similarly, “Do it for your daughter” personalized clinical trials, providing an opportunity to create a world for relatives who come after clinical trial participants, with the hope that they will not have to experience cancer in the same way. As one participant responded,

*“I feel like if I were given the opportunity to make sure that nobody else had to go through that, then I would be willing to do a clinical trial to prevent somebody else from having to go through that.”*

### Quantitative Survey Study

Based on the findings of the qualitative study, the quantitative study was designed to examine which clinical trial myths, truths, and fears are most prevalent; to identify the most prevalent informational, emotional, perceptual, and tactical barriers preventing clinical trial consideration and participation; and to identify preferences for sources of information and communication, among other variables.

#### Participant Characteristics

In total, 257 participants completed the online survey. Based on their answers to survey questions, most participants were stage IV (metastatic) breast cancer patients (46%), were college graduates or had post-graduate degrees (67%), and had a household income of ≥ $75K (58%) (Table 1).

#### Clinical Trial Awareness, Perceptions, and Expectations

**Fig. 1:**
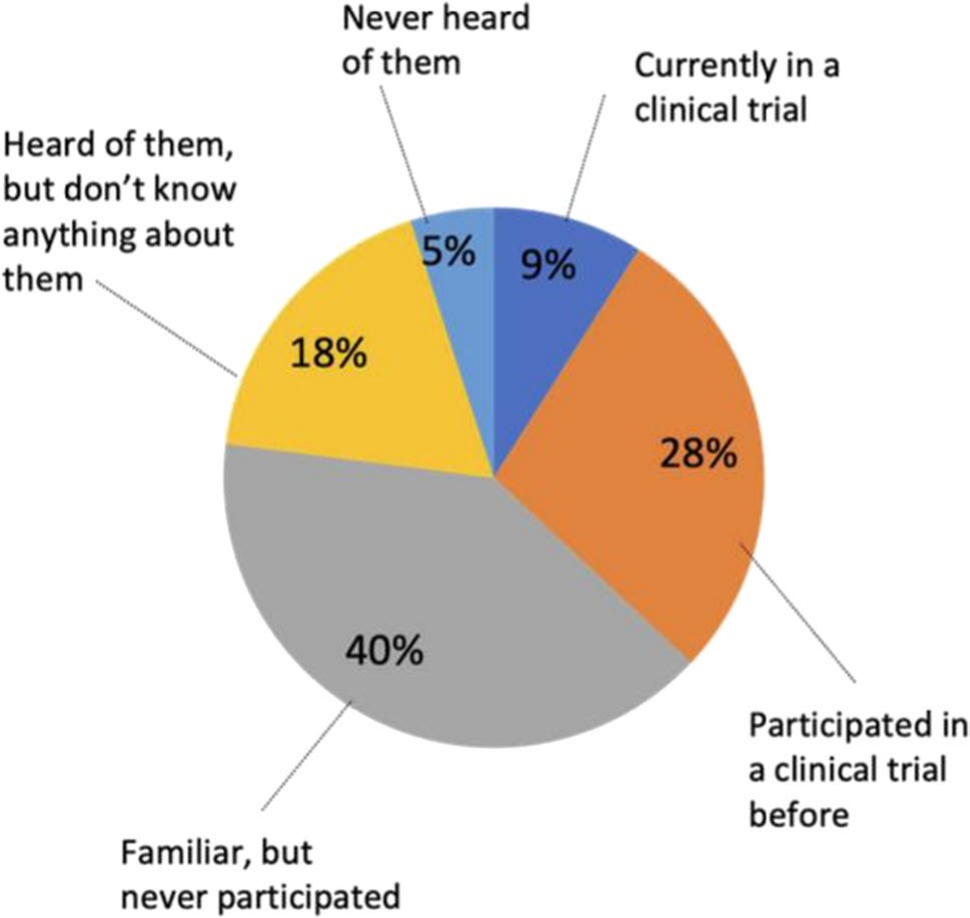
Breakdown of survey participants’ responses to the question, “What is your level of awareness and experience with clinical trials for breast cancer?” (*N* = 257) (Question S21, Supplemental Methods)

Among all 257 survey participants, 40% were familiar with clinical trials but had never participated, while 23% had either not heard of clinical trials (5%) or heard of clinical trials but indicated that they were not familiar with them (18%) (Fig. 1). Participants with metastatic cancer were found to have participated in clinical trials more than those with early-stage cancer (58% vs.19%, *p* < 0.05), and those with higher incomes (≥ $75K) were found to have participated in clinical trials more than those at lower income levels (53% vs.14%, *p* < 0.05).

**Fig. 2:**
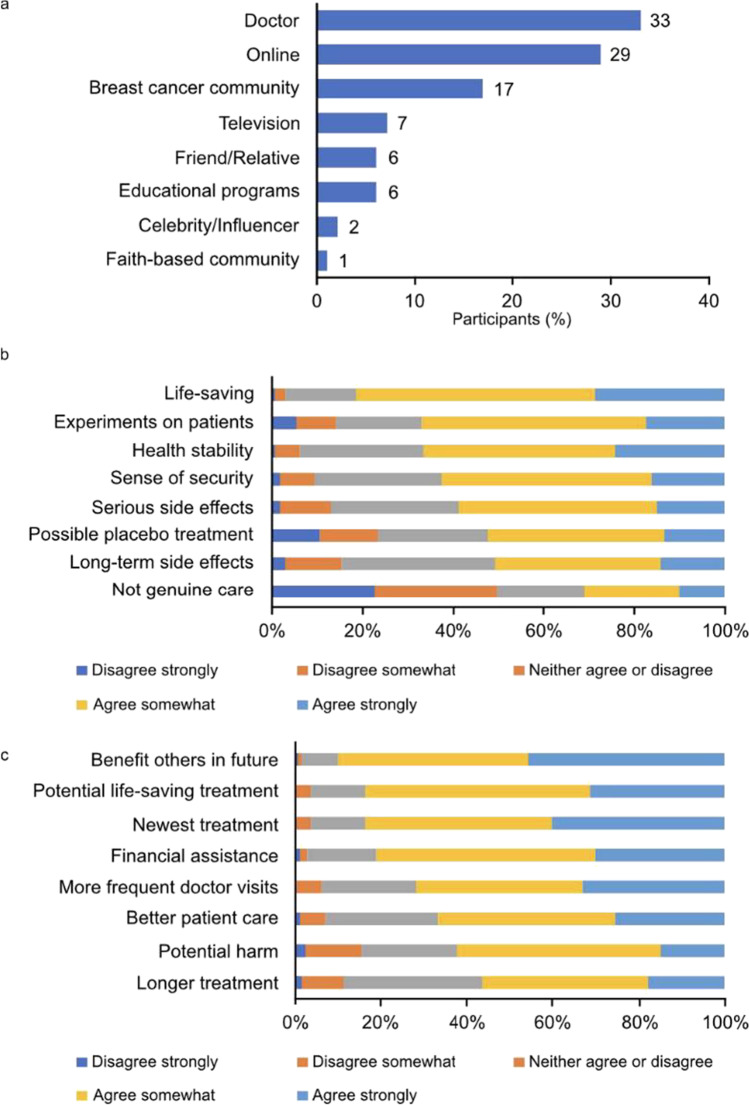
Clinical trial awareness, perceptions, and expectations among participants who indicated that they had heard of clinical trials (*n* = 245). (**a**) Participants reported where they first learned about clinical trials (Question A1, Supplemental Methods). Of note, Doctor includes “Saw a poster or pamphlet for it in a doctor’s office” and “A doctor or healthcare provider mentioned it;” Online includes “Saw an ad pop up online,” “Saw videos online,” “Through my own research via search engines such as Google or Yahoo (not social media),” “Through a post on my social media feed (e.g., Facebook, Instagram, Twitter),” and “Through a breast cancer group or community on social media (e.g., Facebook);” Breast cancer community includes “From another breast cancer patient,” “From a breast cancer support group,” and “From a breast cancer conference;” Educational programs includes “An educational program at a hospital” and “An educational program in a school setting.” (**b**) Participants reported on their perceptions of clinical trials (Question A3, Supplemental Methods). (**c**) Participants reported on their expectations of enrolling in clinical trials (Question A4, Supplemental Methods)

Among the 245 participants who reported they were aware of clinical trials, one-third indicated their HCP as their first source of awareness (Fig. 2a). Approximately one-third listed an online source, defined as online pop-up advertisements, online research, posts on social media, or breast cancer community/groups on social media. The third-most cited source was the breast cancer community, defined as another breast cancer patient, breast cancer support group, or breast cancer conference. When the 245 participants who were familiar with clinical trials were asked about their perceptions related to participating in clinical trials, a majority agreed that clinical trials are lifesaving (81%) and that they bring health stability (67%) and security (62%) (Fig. 2b). However, clinical trials were also associated with experimentation on patients (67%) or treatments that are “not real” (52%). In addition, clinical trials were seen as potentially causing serious (58%) or long-term (51%) side effects. Approximately one-third of participants indicated a belief that clinical trials were dangerous. When asked about expected outcomes of participating in a clinical trial, participants agreed that trials would benefit others (90%), benefit themselves (84%), and provide emerging treatments not otherwise accessible (84%) (Fig. 2c). However, a majority also indicated a risk of potential harm from unproven treatments (62%).

#### Barriers and Motivators to Participation

**Fig. 3:**
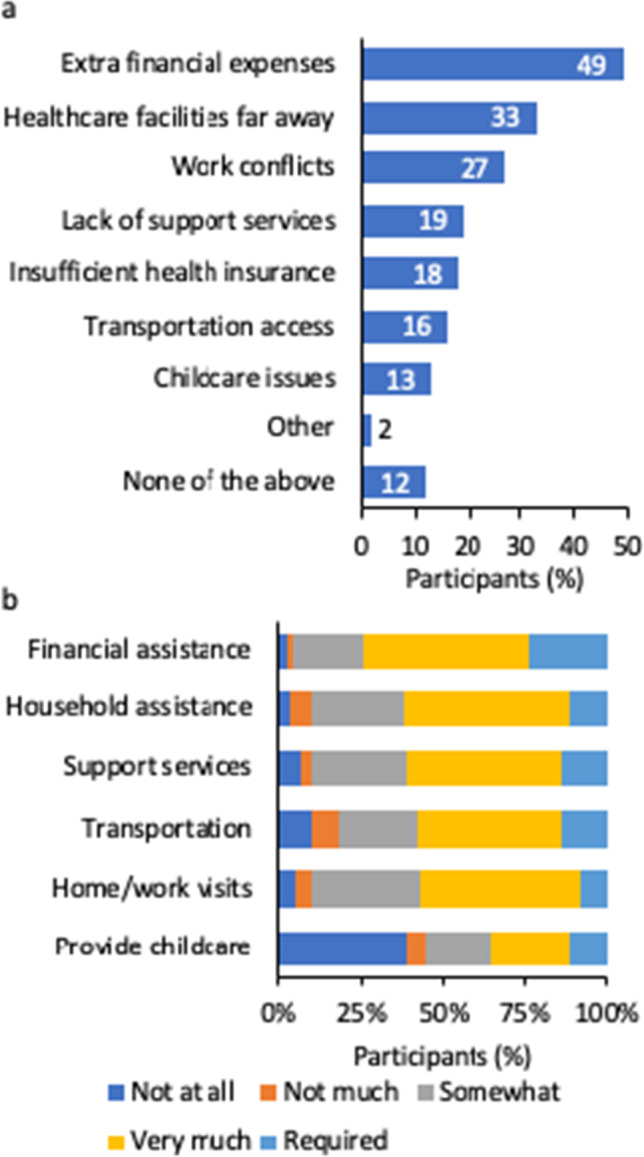
Logistical barriers and motivators for clinical trial participation (*N*=257). (**a**) Participants selected all logistical factors that were considered barriers when asked, “If you wanted to participate in a clinical trial for breast cancer and were selected as a participant, which of the following, if any, do you think would limit your ability to participate?” (Question A16, Supplemental Methods) (**b**) Participants were also asked, “How much, if at all, would any of the following **make it easier** for you to participate in a clinical trial for breast cancer?” (Question A19, Supplemental Methods)

The most commonly reported logistical barriers were financial expenses not covered by the trial (49%), living far away from health care facilities (33%), and interference with work commitments (27%) (Fig. 3a). Difficulty accessing transportation was also cited as a barrier (16%), even when participants lived close to a trial site. In terms of logistical assistance, most participants (74%) indicated that financial assistance would be helpful or required for clinical trial participation, and 24% said it would be required. Other required or helpful factors included household assistance (62%), support services such as mental health counseling (61%), and transportation to the trial site (58%) (Fig. 3b).

**Fig. 4:**
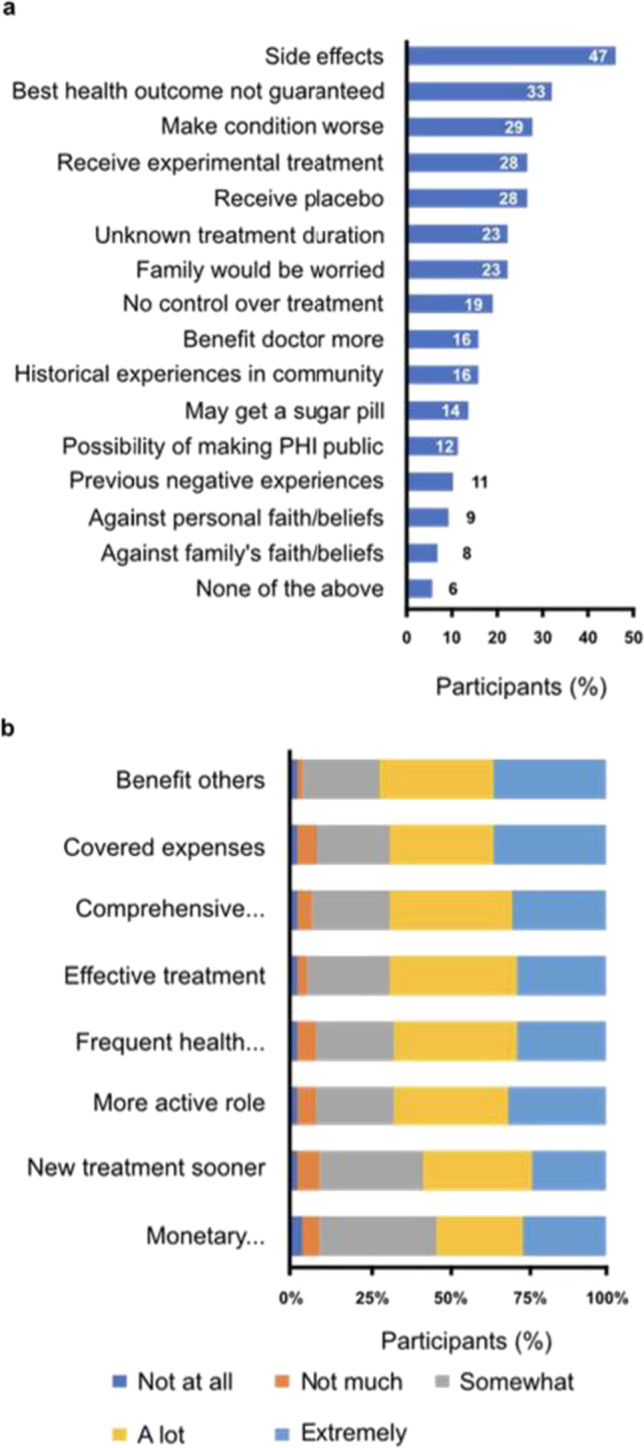
Emotional barriers and motivators for clinical trial participation (*N*=257). (**a**) Participants selected all emotional factors that were considered barriers when asked, “Which of the following, if any, are concerns you have about participating in clinical trials for breast cancer?” (Question A17, Supplemental Methods) (**b**) Participants were also asked, “Below are some potential outcomes from participating in clinical trials for breast cancer. How much do each of the following encourage you to participate in one?” (Question A18, Supplemental Methods)

Emotional barriers to trial participation included previously undiscovered side effects (47%), no guarantees of a best health outcome (33%), and the potential to make one’s condition worse (29%) (Fig. 4a). Of note, 79 participants (30.7%) selected “I may get a placebo” (*n*=71) and/or “I may get a sugar pill” (*n*=35) as a concern, demonstrating that there is a gap in understanding how clinical trials in oncology are designed to compare investigational treatments with standard of care. Concerns regarding not having control (19%), lack of privacy (12%), and overall lack of trust due to past personal negative experiences (11%) were also cited. However, several potential outcomes from participating in clinical trials were reported to encourage patients “a lot” or “extremely” (Fig. 4b). The most common potential outcome that encouraged participation (73%) was “I may help find treatments to benefit others like me in the future.” Other potential outcomes that encouraged clinical participation related to fully covered treatment expenses and more comprehensive health evaluations.

**Fig. 5:**
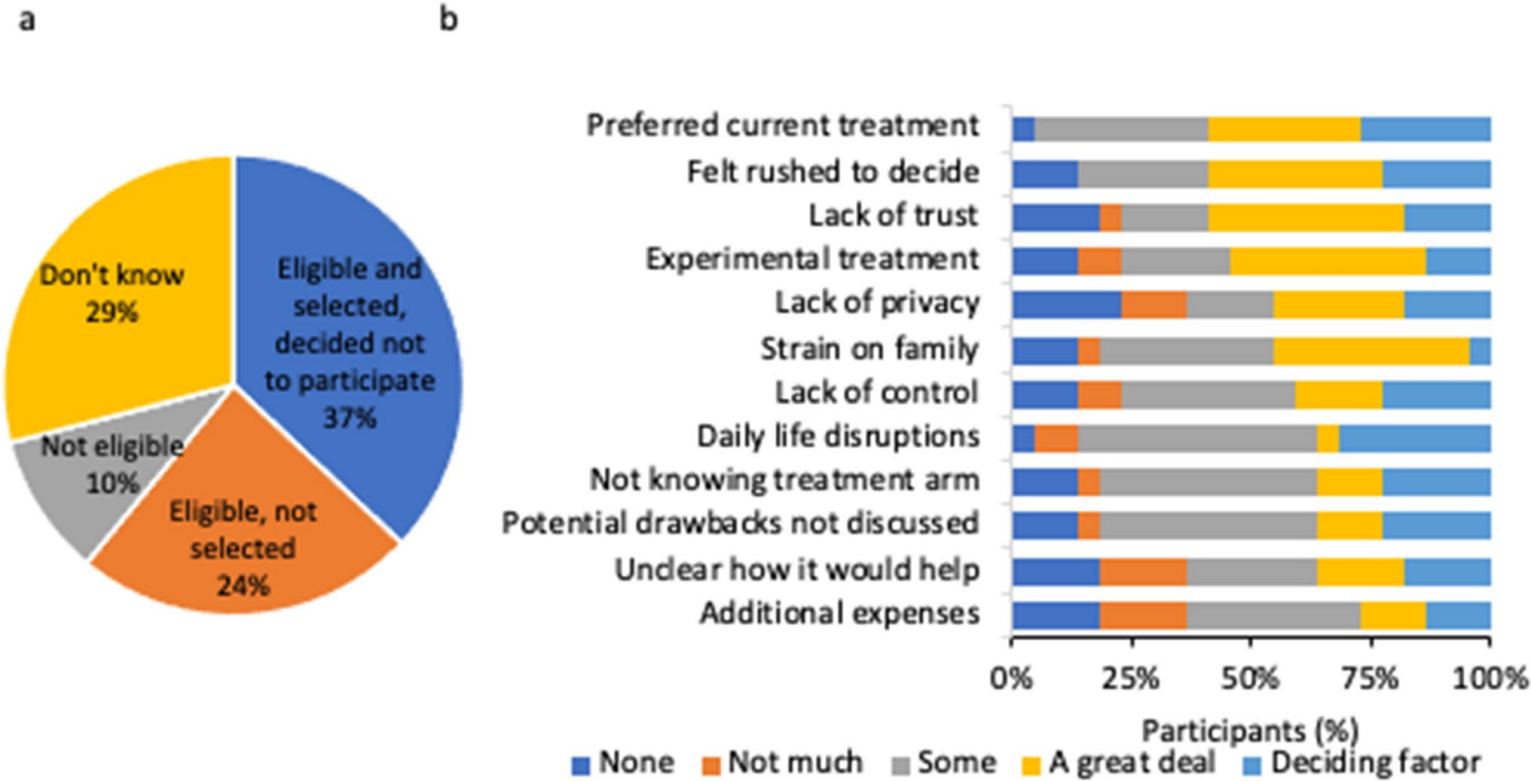
Clinical trial eligibility and participation. (**a**) Decisions on clinical trial participation among participants who had discussed clinical trials but never participated (*n*=59) (Question A13, Supplemental Methods) (**b**) Reasons for not participating among participants who were eligible and were selected to enroll in a clinical trial (*n*=22) (Question A14, Supplemental Methods)

Among 59 participants who reported discussing clinical trials with their HCPs but had never participated, 22 (37%) reported that they had decided not to participate even though they were eligible for a clinical trial for breast cancer and had been selected for inclusion (Fig. 5a). The most-cited reasons (either deciding or influencing factors) were a preference for current treatment (59%), not having a well-established relationship with their HCP or person who introduced the possibility of participating in a trial (59%), a feeling of being rushed or pressured into a decision (59%), or the opinion that experimental treatment was not yet needed (55%) (Fig. 5b).

#### Relationships with Health Care Providers and the Health Care System

**Fig. 6:**
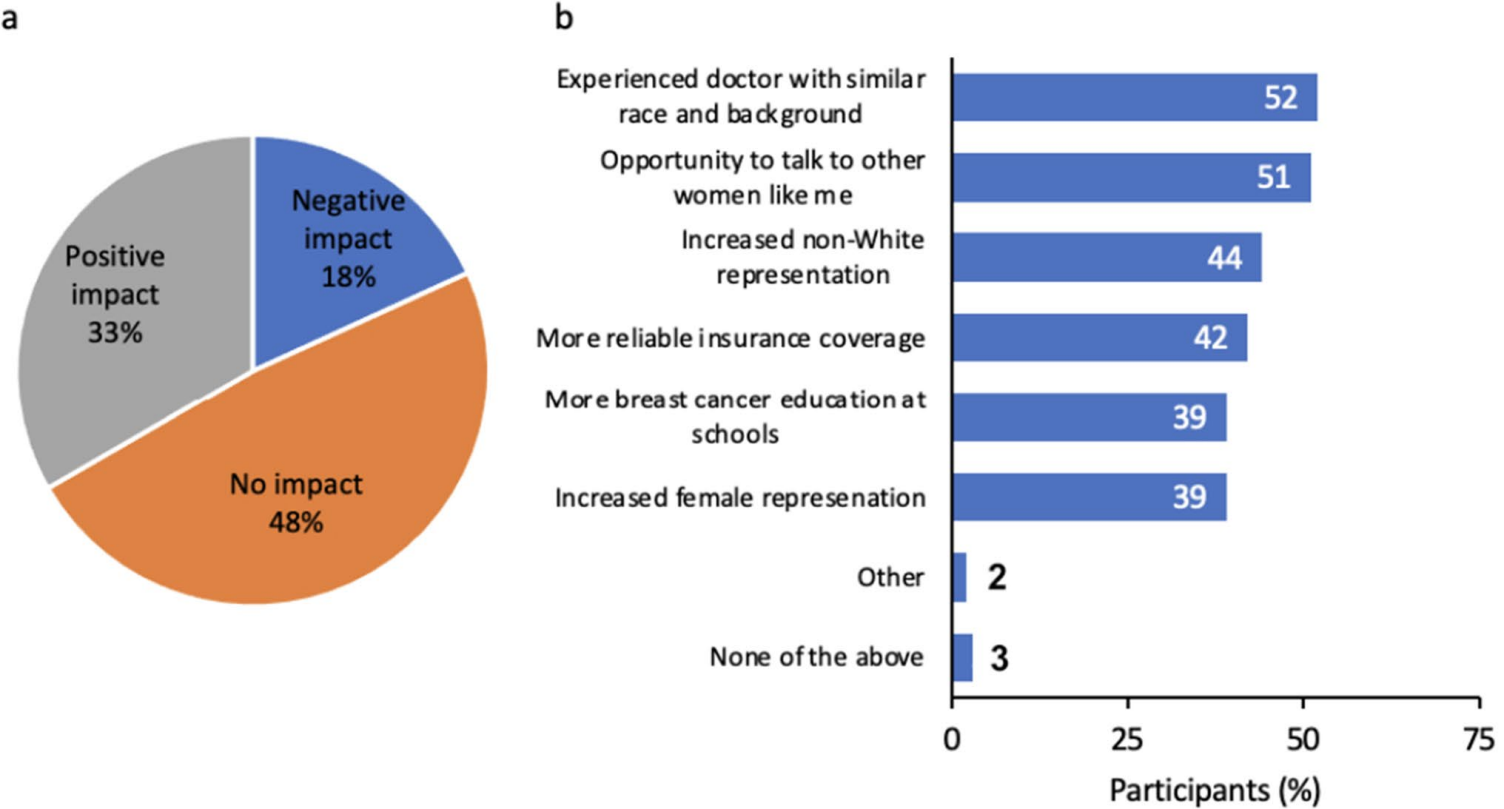
Relationships with healthcare providers and the healthcare system. (**a**) Participants were asked, “What impact do you think your race has on the quality of the medical care treatment you receive for breast cancer?” (Question C1, Supplemental Methods) (**b**) Additionally, participants were asked, “Which of the following, if any, do you feel would create a more equitable healthcare experience for you the most?” (Question C2, Supplemental Methods)

Approximately half of the participants indicated a belief that their race has no impact on the quality of medical care they receive; those who believed their race has a negative impact were more likely to be lower/middle-income and early-stage patients (Fig. 6a). Nearly half of participants (48%) reported perceiving their doctor as Black or African American, and most (76%) reported seeing a female doctor for breast cancer care. Among the 96 participants who had been in a clinical trial, most had Black (75%) and/or female (88%) doctors. When all participants were asked what they felt would create a more equitable health care experience, most wanted assurance that their doctor had experience treating patients with races and backgrounds similar to theirs (52%), and the opportunity to talk to other women with similar races, backgrounds, and diagnoses (51%) (Fig. 6b).

#### Discussing Clinical Trials with Health Care Providers

**Fig. 7:**
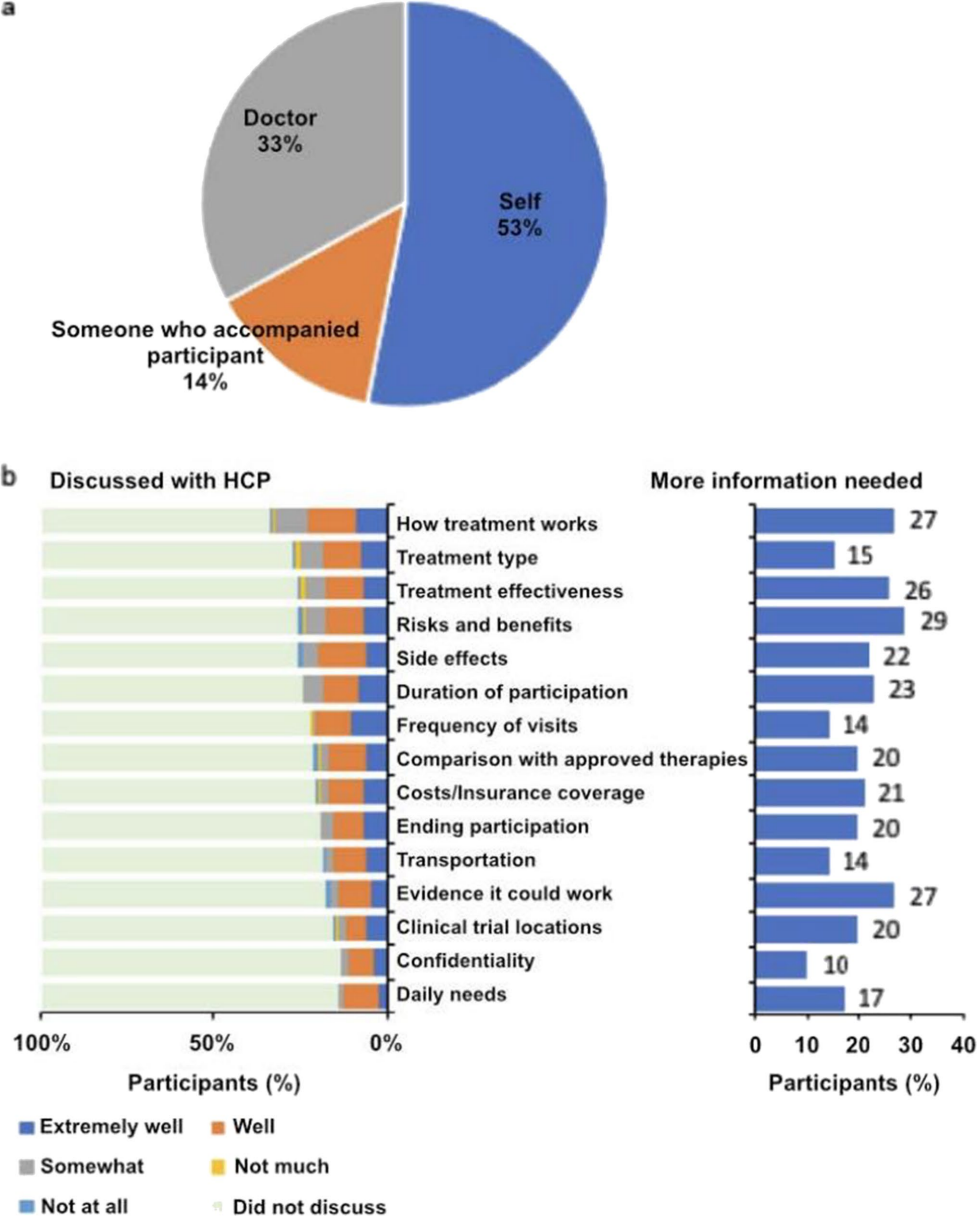
Discussing clinical trials with health care providers (HCPs). (**a**) Among participants who reported discussing clinical trials with an HCP (*n*=155), the following question was asked: “During the **first** time you talked to your doctor about possibly participating in a clinical trial for breast cancer, who brought it up?” (Question A7, Supplemental Methods) (**b**) These participants were also asked about which topics were discussed: “When you talked to your doctor about possibly participating in a clinical trial for breast cancer, which of the following topics did you talk about?” (Question A9, Supplemental Methods). Among the participants who reported that they discussed a topic, they were asked to report how well they understood the topic (left, Question A10, Supplemental Methods). They were also asked, “Which of the following topics would you have wanted more information about?” (right, Question A11, Supplemental Methods)

Among all participants, 155 (60%) reported that they had discussed clinical trials with their HCPs at least one time; 47 (30%) had discussed them one time, 83 (54%) two times, and 25 (16%) three or more times. The participants themselves and/or loved ones attending the health care visits were more likely than the HCPs to initiate the discussions (67% versus 33%, Fig. 7a). Among the 155 participants who had discussed trials with their HCPs, 26% discussed treatment efficacy, potential risks and benefits, and potential side effects (Fig. 6b, left). In all cases, at least two-thirds of participants indicated that they understood each topic either “well” or “extremely well” (Fig. 7b, left). However, almost one-third (29%) of participants indicated a need for more information about risks and benefits to their personal health (Fig. 7b, right).

#### Community Engagement and Resources

**Fig. 8:**
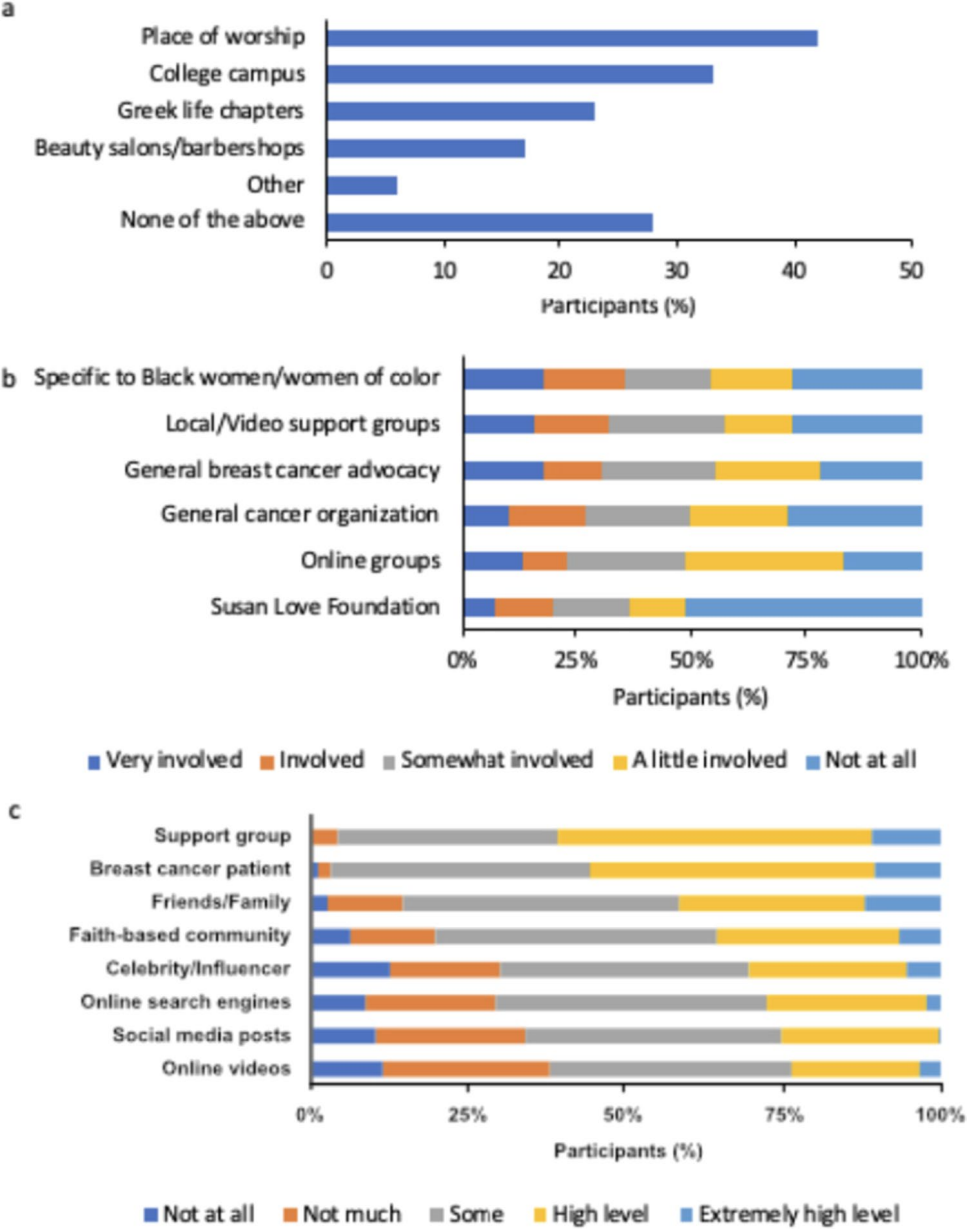
Community engagement and resources among all quantitative study participants (*N*=257). (**a**) Participants were asked, “Through which of the following community-based touchpoints/networks would you be open to receiving information about clinical trials for breast cancer?” Participants could select all responses that applied (Question A21, Supplemental Methods) (**b**) Participants were asked, “Since the breast cancer diagnosis, how involved, if at all, have you been with any of the following?” (Question D1, Supplemental Methods) (**c**) Participants were asked, “When it comes to receiving accurate and reliable information about clinical trials for breast cancer, how much do you trust each of the following sources?” (Question A20, Supplemental Methods)

Participants indicated that they were open to church or other places of worship as community-based sources of information on clinical trials (42%), with college campuses (33%) and Greek life chapters (23%) also preferred sources (Fig. 8a). When asked if they were involved in any breast cancer community organization, 66% of participants indicated a high level of involvement. Involvement was even higher among those who reported to have participated in clinical trials (89%), those who reported to have metastatic cancer (82%), or those with household incomes ≥ $75,000 (83%). A third of participants (36%) indicated that they were involved with a breast cancer organization specific to Black women or women of color (Fig. 8b). Participants who were involved/very involved in the breast cancer community primarily engaged through breast cancer organizations and support groups. Most participants had high levels of trust in HCPs and breast cancer support groups as sources of information on clinical trials, as well as in breast cancer conferences or other breast cancer patients (Fig. 8c). Those active in the breast cancer community showed significantly more trust in all sources of information about clinical trials (Supplemental Fig. 1).

#### Patient-Directed Electronic Data Platform

Among 106 survey participants who utilized the Ciitizen data platform, 61 had clinical records available for analysis. Although not precise, participants’ self-reported knowledge of their stage of cancer was reflected in Ciitizen records approximately 50% of the time (Supplemental Fig. 2). It is important to note that reasons for discordance between records and survey responses could not be determined based on the available data. However, some of the discordance may be due to the comparison of self-reported information from a cross-sectional cohort with medical records-extracted longitudinal information that captures stage across time, including patients who have experienced relapse or progression of disease. Another source of potential discordance is the manner in which HCPs communicated staging information (e.g., some patients only know “early stage” versus “late stage” without more granularity). The most common molecular types of breast cancer were HR+/HER2- (59%) and triple-negative (23%) breast cancer. At least one comorbid condition was reported in 77% of participants, with anxiety (33%), hypertension (33%), and asthma (21%) reported as the most common comorbidities. At least one treatment regimen was reported for 79% of patients, including chemotherapy (67%), hormonal therapy (44%), targeted therapy (29%), and immunotherapy (5%). In addition, a vast majority (75%) of participants had a reported surgery and two-thirds (64%) had a reported non-surgical procedure. At least one adverse event was reported in 67% of participants, with fatigue (31%), nausea (20%), and pain (20%) being the most common.

## Discussion

This mixed methods research specifically engaged Black breast cancer patients in order to better understand their perceptions of clinical trials and the factors that influence their decisions regarding participation.

For the quantitative study, most participants responded that they were familiar with clinical trials, with the most common sources of first awareness being their HCPs or online sources such as research, advertisements, or social media. Direct interaction with other breast cancer patients, breast cancer support groups, or breast cancer conferences were also commonly cited as sources where patients first learned about clinical trials. However, a misunderstanding of clinical trial design, specifically a misconception that participation meant that participants would not receive any treatment if assigned to the control group (i.e., that they would receive a placebo or “sugar pill”), was a clear barrier to participation.

The vast majority of patients expected clinical trials to help identify potential lifesaving treatments that would benefit others in their community. They also agreed that they themselves would benefit from clinical trials, such as by having access to the newest medications or treatments they otherwise could not afford. Participants expressed concerns about the logistical barriers to trial participation, including financial expenses not covered, transportation issues, and interference with work commitments. Of note, the majority of participants who indicated that household assistance such as meals, cleaning, or daily care would be helpful or required for participation had not participated in clinical trials before. Thus, when appraising barriers to participation, it is important to consider concerns raised by those with real-world experience with clinical trials, especially considering that the decision to participate in a clinical trial is likely influenced by the past experiences of friends, relatives, or other community members.

Underlying the emotional barriers to trial participation were participants' fears of the unknown, including potential harm from unproven treatments, longer treatment periods for their condition, or possible worsening of their condition. Study participants specifically referred to past abuses, including the Tuskegee syphilis study in which African American men were denied the standard treatment for their condition [44] and the story of Henrietta Lacks, whose cervical cancer cells were collected and cultured without her knowledge or permission. The resulting immortalized cell line went on to facilitate extremely lucrative discoveries, although Lacks’ family received no financial compensation [45, 46]. These historical traumas have left emotional scars within the Black community that continue to be a barrier to clinical research participation. Recent discussions regarding medical mistrust have pointed out that such fears are a substantiated reflection of the healthcare system, not of individuals; thus, addressing medical mistrust must occur at the systemic level [47, 48].

Despite study participants’ fears of the unknown, most reported that they trusted and were satisfied with their HCPs; however, 18% believed that their race had a negative impact on the quality of their medical care. Those who had participated in a clinical trial were more likely to have a Black and/or female doctor, showing a potential link between trial participation and relationships between HCPs and patients of similar racial and/or gender backgrounds. For many participants, more equitable health care meant HCPs experienced in treating Black breast cancer patients, opportunities to talk to breast cancer patients like them, and increased non-White female representation within their care team. This also relates to medical mistrust, as increased representation of Black female oncologists, nurses, counselors, and other specialists can improve communication to address patients’ concerns.

Although participants reported high levels of trust in their HCPs, when reporting on past discussions of clinical trials with their HCPs, almost a third felt that information regarding risks and benefits was lacking. More than a third of patients who were eligible and selected to participate in a clinical trial chose not to participate because they did not have a well-established HCP relationship or felt rushed into making a decision. This indicates a need for HCPs to be more proactive in introducing and managing clinical trial discussions well before patients may be considered for a trial, in order to provide education and to overcome any emotional barriers.

Community and membership-based groups such as churches, colleges, or Greek life were identified as preferred sources of information on clinical trials. Participants expressed trust in breast cancer support groups (64%) or other breast cancer patients (59%) almost as much as in their HCPs (66%). They also expressed high levels of trust in receiving information from friends and family members (46%). Since receiving “sugar pills,” “not getting the real treatment,” or “being experimented on,” were indicated as perceived risks associated with breast cancer clinical trials, communications with friends or family members who also hold these perceptions may compound misconceptions. Culturally tailored education efforts to explain how clinical trials work are clearly needed to ameliorate doubts for Black women. Engaging Black women’s immediate support networks is also important, as these play a crucial role in decision-making. Resources such as plain language summaries to explain clinical trials in an understandable way can also be a valuable tool for patients when discussing with family and friends the option of participating in a clinical trial [49].

Participants, particularly those involved in the breast cancer community, expressed a high level of trust in breast cancer support groups. The fact that 40% of survey respondents were referred to the survey by advocacy groups indicates that trusted communities may be effectively used as a valuable resource for education on clinical trials, as well as for information regarding what studies are available. These organizations provide opportunities for cancer centers and HCPs to build relationships and implement community-based research to increase access and participation in clinical trials [35, 50].

Determining eligibility for clinical trials primarily relies on physician and patient knowledge and research. With the expanding use of electronic health records, data from medical records, billing records, and other clinical and administrative data can provide a more comprehensive description of a patient’s cancer diagnosis, treatment, and follow-up [51]. To overcome implicit biases and improve access to clinical trial participation, it is critical that all eligible patients be asked to participate in clinical trials. If this occurs, diverse participation in clinical trials is more likely to be achieved [35, 36]. In our research, the Ciitizen platform enabled access to clinical data beyond what was self-reported by patients, providing comprehensive information for use in clinical trial matching. This collaboration among breast cancer advocacy groups, academic institutions, and non-profit organizations with a patient-controlled data platform illustrates an effective new strategy for promoting clinical trial awareness and participation for all patients.

This research and its findings speak to the emotional barriers—namely ideological fear and earned medical mistrust—around clinical trials in the Black breast cancer community. Addressing this fear via trusted messengers can enable shifts in attitude towards clinical trials for this population and is a pre-cursor to the work of addressing logistical, educational, and systemic barriers.

## Limitations

The findings of this study should be considered in the context of some limitations. The recruitment strategy introduced bias towards higher education and income level of participants, as well as a higher level of clinical trial participation and influence of perceived race or ethnicity of the participants’ HCPs. Future efforts should expand the sample size to ensure comprehensive representation of Black women, including those with lower household incomes, those who live in rural areas, those with stage I-III breast cancer, and non-college-educated Black women. As well, the absence of a control group for comparison limits our conclusions regarding opinions unique to Black women.

## Conclusion

In conclusion, this research provides valuable insights into the factors underlying why Black women choose not to participate in clinical trials. Our findings highlight the importance of building trust, providing education, and resolving misperceptions for Black women diagnosed with breast cancer to empower them to make fully informed decisions when considering participating in clinical trials. They also highlight the importance of fully utilizing strategies to identify all eligible patients and ask them to participate in clinical trials.

### Supplementary Information

ESM 1 (DOCX 589 kb)
