# Supplementary material for: Increasing Clinical Trial Participation of Black Women Diagnosed with Breast Cancer
Source: J Racial Ethn Health Disparities. 2023 Jun 14;11(3):1701–17. doi: 10.1007/s40615-023-01644-z (PMC11101578; doi:10.1007/s40615-023-01644-z)
Supplement: Supplementary file 1 — (DOCX 589 kb) [file 40615_2023_1644_MOESM1_ESM.docx]

**Supplement to:** Increasing Clinical Trial Participation of Black Women Diagnosed with Breast Cancer

[**Supplemental Methods**](#_uob5pjyi3spw) **2**

[Qualitative Survey Discussion Guide](#_wl7gtn3y31xi) 2

[Section A: Introduction](#_566q5tyqbrdc) 2

[Section B: Her Story - Diagnosis, Treatment Plan, Doctor Trust and Quality of Care](#_32ohcjp0uef6) 2

[Section C: Self-Definition](#_i0h5bb1jnxwy) 3

[Section D: Understanding of Clinical Trials](#_9l32wwy5ra3o) 5

[Section F: Unmet Needs Relative to Participation](#_xr2jq98ove0g) 6

[Section G: Recruiting Messaging and Tactics](#_pla5cpnk5631) 7

[Section H: Wrap Up](#_ffe2cfc67hgr) 8

[Quantitative Questionnaire](#_exlx8196vk02) 9

[Demographics, Clinical Characteristics, and Eligibility Criteria](#_633z84mvpd68) 9

[Clinical Trials Perceptions - Among Aware/Participated](#_tcjf7x47p3bw) 21

[Clinical Trial Conversations - Among Who Discussed with Health Care Provider](#_ndzamm2th19s) 24

[Clinical Trial Barriers and Motivators - Among ALL](#_l0ilgxkvga4m) 28

[Relationship with Health Care Providers - Among All](#_i2qfop3px57q) 31

[Racial Impact - Among All](#_uiqt6cso09hr) 32

[Demographics](#_vpfeacv755k4) 33

[**Supplemental Figures**](#_j5urqzy2jq9m) **37**

[Supplemental Fig 1](#_aoc1ymr09cl) 37

# Supplemental Methods

## Qualitative Survey Discussion Guide for Focus Groups and Individual Interviews

### Section A: Introduction

**Objective:** Introductions, disclosures, and icebreaker

**Estimated time:** 1 minute

- Introductions and subject of discussion: Breast Cancer and Clinical Trial Research
- LRW, the firm conducting this research, is an independent market research agency and consultancy. The research is sponsored by a pharmaceutical company.
- We will comply with all laws that protect your personal data.
- Compensation for participation in the study is as indicated in your invitation.
- The purpose of conducting this study is not to sell or promote anything to you, but rather to understand your views.
- You may withdraw from the market research at any time, and if you wish to not answer a question or want to withhold information at any point during the survey, you may withdraw from the conversation at any time.
- There are some people watching and listening, but they too will maintain your confidentiality.
- I am a neutral moderator and there are no right or wrong answers
- **Icebreaker:** What is the first thing you will do when you are freed from covid?

### Section B: Her Story - Diagnosis, Treatment Plan, Doctor Trust and Quality of Care

**Objectives:** Identify knowledge gaps in breast cancer, breast cancer care. Understand the superwoman theory and how it impacts health. Evaluate how cancer makes them feel as a black woman and their impact on managing their responsibilities.

**Estimated time:** 15 minutes

1. How did you **first learn** you had breast cancer?
   1. Had you suspected anything was wrong or was it a total shock?
   2. What kind of doctor diagnosed you? Do you remember the words he/she used?
2. What was your **treatment plan**?
   1. How was it explained to you?
   2. How did you feel about your treatment plan? Did you **trust** that this was the right plan? Why or why not?
   3. What conversations did you have about your treatment plan? With whom, how many and for how long?
   4. What information sources did you consult about your treatment plan?
   5. Was there anything that **scared** you about your treatment plan? Did you raise these concerns with your medical team? How did they address these concerns?
3. Were there **options for discussion in your treatment plan**? For example, there may have been choices of different chemotherapies, or choices of what type of surgery to have.
   1. How did it make you feel to have options?
   2. Do you feel that the options were well explained to you?
4. Let’s talk some more about your **medical oncologist**.
   1. How do you feel when you speak to him/her?
   2. How confident do you feel in his/her recommendations?
   3. How well does he/she explain things to you?
   4. Do you feel like he/she has taken the time to know you as a person?
   5. How comfortable do you feel asking questions? Do you worry about “looking dumb” or do you just say what is on your mind?
   6. Do you trust him/her?
   7. If he/she needed to refer you to see another doctor, how comfortable would you feel with this referral?
      1. PROBE on rationale. (Note: this is pertinent to trials as it is a referral to see dr who manages trials)
5. Are there **other members of your medical team** whom you feel more comfortable with, than your medical oncologist?
   1. PROBE: oncology nurse. Do you feel like it is easier to ask questions to the nurse, that he/she spends more time with you. Do you relate more to the oncology nurse? (PROBE: gender dynamics, nurses are usually female)
   2. Do you trust her?
6. Overall, how do you feel about the **quality of care** you have received?
   1. Do you feel like anything would have been different if you were White?

### Section C: Self-Definition

**Objectives:** Understand her role as a caregiver and provider for her family, and the emotions she experiences. *Is her role in life is at odds with being a breast cancer patient?* She has **power** because she provides; breast cancer chips away at this power. It compromises her ability to provide, and makes her feel vulnerable as undergoes treatment with side effects and outcomes that are **out of her control**.

We hypothesize that she perceives clinical trials as chipping away at her power even moreso than conventional cancer treatments, since she must surrender even more to unknown side effects and unknown outcomes. This section of the discussion guide uncovers how she sees herself so that we can understand psychological barriers that come into play vis-à-vis clinical trials.

**Estimated time:** 20 minutes

1. Tell us about your life.
   1. How many children do you have?
   2. Are you married?
   3. What is your household like? Who lives in your home with you?
   4. Are you the financial provider for your family? Did this change DURING your breast cancer diagnosis? AFTER your breast cancer diagnosis?
   5. What kind of work do you do?
   6. How would you rate your overall **stress** level? (scale of 1-5)
2. What is the **theme song** to your life? Why?
3. Do you feel like a **Superwoman**? What color is your cape?
   1. Is being a Superwoman a burden?
   2. Or does it give you power?
   3. Or both?
4. Let’s think about a **typical day** in your life **PRIOR to breast cancer**. PROBE:
   1. Needs of kids
   2. Your work (inside the home and outside the home)
   3. Your significant other
   4. Free time for yourself?
   5. How would you typically feel? Totally exhausted? Overwhelmed? Stressed?
   6. Where did you get your strength?
5. Does having breast cancer come into **conflict with being a Superwoman**?
6. How did your **typical day** change **DURING treatment** for cancer? PROBE:
   1. Did expectations of you and what you could do for others change
   2. Did anyone step in to help? PROBE.
   3. Were you able to fit in time to rest?
7. (If post-treatment) What is a **typical day** in your life like NOW?
   1. Are expectations back to where they were prior to breast cancer?
      1. Do you feel like you can still do as much as you did prior to breast cancer? Or do you feel permanently changed?
8. Let’s talk about a **few words** and what they mean to you:
   1. Bad ass
   2. Power
   3. Control
   4. Vulnerability
   5. Guilt
9. Do you feel like you have **choices** in your life? Or do you feel locked into things as they are?
10. If you could **wave a magic wand** and make you some of your responsibilities go away, what would you choose? Why?
    1. What responsibilities would you definitely want to hold onto?
    2. Would you ever give it up and go live at a spa on a remote island?
    3. Are you afraid to take the cape off?
11. What keeps you up at night? Did that change after cancer?
12. Do White women wear a cape? What color is it?

### Section D: Understanding of Clinical Trials

**Objective:** Uncover and seek to understand awareness, perceptions, and beliefs that drive the genuine emotional barriers to clinical trial participation

**Estimated time:** 20 minutes

1. We are now going to shift to a different topic. **Medical research or also called clinical trials. Do you know what it is?**
   1. What do you think about medical research?
   2. Are there any particular examples that come to mind?
   3. How important do you think it is?
   4. What is good about it? What, if anything, concerns you? PROBE: privacy, being a guinea pig, selling the data, placebo effect.
   5. Do you believe **Black people** have been mistreated in medical research?
      1. Tell me about what you know and how it makes you feel?
      2. How do you know about the history? How did you find out about it?
      3. Do you think the things that happened in the past are still happening today?
   6. Do you know of anyone personally that participated in medical research? Was their experience good or bad? Why?
2. What about **medical research for breast cancer**?
   1. Do you ever think about how research could have impacted your treatment?
   2. Do you think there is a need for drugs that work better than the ones we currently have for breast cancer?
3. What about **clinical trials**? What comes to mind?
   1. How did you first hear about clinical trials?
   2. Do you know of anyone who is in a clinical trial? Have you ever talked about it with anyone?
   3. What is your **understanding** of how trials work for breast cancer? PROBE:
      1. Does everyone get the same drug? Is there a placebo?
      2. What patients are the right fit? PROBE: age, ethnicity, cancer stage, socioeconomic status.
      3. Are clinical trials more risky than conventional treatment? Why?
      4. What concerns would you have about participating in a clinical trial for breast cancer? PROBE: Placebo, can I stop the trial if I want to, a lot paperwork, insurance headaches, quality of care, what if trial doesn’t work, integrity of my doctors/ethics/are they doing the right thing
   4. I am going to show you some information defining what a clinical trial is and ask for your reactions.
      1. Clinical trials are research studies meant to evaluate a medical, surgical, or behavioral intervention in humans.
      2. Clinical trials are an important step in discovering new treatments for breast cancer and other diseases.
      3. They are the primary way that researchers find out if a new treatment—like a new drug, diet, or medical device (for example, a pacemaker)—is safe and effective in people.
      4. Often a clinical trial is used to learn if a new treatment is more effective and/or has less harmful side effects than the current standard treatment.
      5. They help determine new ways to detect, diagnose, and reduce the risk of disease. Clinical trials also help researchers and doctors decide if the benefits of a new treatment outweigh the side effects.
   5. Reactions to stimulus
      1. What did you learn that is new?
      2. What do you have questions about?
      3. With this information, does it change any of your opinions?
   6. Do you feel that you are the right fit for a clinical trial? Why or why not? How do you know if you are a good fit?
   7. Are clinical trials for Superwomen? Why or why not?
   8. How does being in a clinical trial compare to conventional treatment in terms of the impact on your life? PROBE: time consuming, travel, missing work, different doctors, unknown risks, potential to kill me, additional stress, fear of the unknown, confusing information
   9. Do you have any fears about clinical trials?

### Section F: Unmet Needs Relative to Participation

**Objective:** Assess the unmet needs that must be addressed in order to drive participation in clinical trials. Understand and prioritize the barriers to participation (informational, perceptual, emotional, and logistical) towards the development of relevant messaging.

**Estimated time:** 30 minutes

1. We are trying to help Black women feel more comfortable about participating in clinical trial research because we believe we need to make drugs that work better on Black women’s bodies. We know there is a lot of fear around this as we have talked about though out our time together today. We need your help to figure out what would make you and other Black women feel more comfortable to participate. Some of the things we think matter for a Black woman to want to participate in a clinical trial include:
   1. Who talks to you—Who should talk to you? Would you feel better if it was coming from a Black health professional? Male or female?
   2. What words the person uses—What would you like to hear or not hear?
   3. What you would want to know—Exactly what would you want to know?
   4. Why the doctor is even bringing up the idea of a trial—some patients think trials are only used as a last resort. What do you think about that?
   5. Why a trial could be a good option for you—would knowing all of the options available to you be helpful?
   6. Knowing information about the drug/therapy—what would you want to know? Would it help if you met some other patients in the trial?
   7. Fear of the unknown—what would you be afraid of and why? Probe.
   8. Interrupting your work (home and job)—what would worry you?
   9. The impact on your family—would you worry about not being able to take care of your family?
   10. Side effects—do you think the side effects in a clinical trial would be different that the ones you experienced (are experiencing) with the chemo you are familiar with? Probe.
2. What would make you the most comfortable in that situation? What would make you uncomfortable? Probe on trust.
3. What words would encourage you to participate?
4. Would you feel better about participating in a trial if you knew that the research could help your children or other family members?
5. How and when would you talk to your family about participating in a clinical trial?
   1. How would you explain it to them?
   2. Would you let them help you with the decision-making?
   3. Who in your family is your confidante/go-to-first person?
6. Do you think your family would be for or against clinical trials? Why or why not?
7. If you decided to do a trial, would your family support you?
8. Did you know that you get better quality of care on clinical trial and that people on trials live longer? Does knowing that make you feel more comfortable?
9. Is there anything else you can think about that we haven’t covered here that makes you feel what you feel about clinical trials? Probe.

### Section G: Recruiting Messaging and Tactics

**Objectives:** Understand the disconnect from current recruiting tactics, information, and messaging. Measure reactions to current language and terminology. Get feedback on stimuli.

**Estimated time:** 33 minutes

1. Have you ever googled clinical trials?
   1. What did you find?
   2. Did you find the information you were looking for?
   3. Do you know what websites you found/explored? Probe.
2. What’s the best way to help you understand how the clinical trial would work? Would you want
   1. a website?
   2. a pamphlet to read?
   3. someone to talk to? Who?
   4. a video?
   5. an app on your phone?
3. Have you ever read a brochure about clinical trials?
   1. From whom/where did it come?
   2. Did you learn anything? Was it helpful? Probe.
4. Would you trust a celebrity to talk to you about clinical trials?
   1. Would you believe them?
   2. When was the last time you bought something based on a celebrity recommending it?
5. We are going to show you a few things so you can give us some feedback. We want you to tell us if any of these things make you feel good about participating in a clinical trial. Get thoughts on each one and then ask them to compare, evaluate if any make a difference. Probe
   1. Concept Statements
      1. You can make a difference
      2. Do it for your daughter
      3. Everything has been in a trial
      4. Best Care
      5. Breastie Shero
6. So now that we have had this conversation, how do you feel about participating in clinical trial research? Probe.

### Section H: Wrap Up

**Estimated time:** 1 minute

## Quantitative Questionnaire

### Demographics, Clinical Characteristics, and Eligibility Criteria

| [Intro] | | |
| --- | --- | --- |
| **- Show to all.** | |  |
| DT_Intro. | We would like to invite you to participate in a survey about clinical trials for breast cancer. Your perspective is extremely important to us.  Please take a moment to complete this survey. All responses will be kept confidential.  **Before you start, note that…**  ● This survey will take approximately **25 minutes** to complete.  ● This survey can be completed using a **computer or a tablet or a smartphone**.  ● If for any reason you click out of the survey window or need to pause, you can come back to continue the survey by clicking on the link in your invitation.  ● Please do not use your browser's forward and back buttons. Instead use the 'Next' button to move through the survey.  ● Material, an independent marketing research firm, will conduct this survey on our behalf. For more information on how we use your responses please see Material’s Terms of Use and Privacy Policy.  Thank you! |  |

| [Consent] | | | |
| --- | --- | --- | --- |
| **- Show to all.** | | |  |
| S1_Consent. | Thank you for considering taking part in our research project.  You are invited to participate in an online survey about health and medical care led by a collaborative group of researchers and advocacy partners. The objective of this study is to better understand the needs of people like you. The information learned in this survey will be used to inform the medical community in an effort to improve accessibility to services.   1. We promise to protect your privacy and treat the information you give us as confidential. 2. The information you provide will be used only for research purposes. 3. We will not release your personal information to any third party without your consent. 4. We will never try to sell you anything and we will never sell your personal data to anyone. We are market researchers interested only in your opinions and behavior. 5. We combine your survey responses in a given survey with the responses of all others who participate and report those combined responses to the sponsor that commissioned the study. 6. Your decisions about participating in this study, responding to specific questions, or discontinuing participation will be respected without question. Your participation is completely voluntary and you may discontinue your participation in the survey at any time. 7. Additional relevant information and options are available to you at https://lrwonline.com /privacy-policy/ and https://lrwgroup.com /yourdatayourrights/   Do you agree to participate in this study? |  |  |
|  | *Select one response.* |  |  |

|  | **DEFAULT ORDER.** | |  | |  | |
| --- | --- | --- | --- | --- | --- | --- |
|  | 1. | I agree to participate | |  | |  |
|  | 2. | I do not agree to participate | |  | |  |

| [Gender] | | | |
| --- | --- | --- | --- |
| **- Show to all.** | | |  |
| S2_Gender. | What gender identity do you most identify with? |  |  |
|  | *Select one response.* |  |  |

|  | **DEFAULT ORDER.** | |  | |  | |
| --- | --- | --- | --- | --- | --- | --- |
|  | 1. | Male | |  | |  |
|  | 2. | Female | |  | |  |
|  | 3. | Transgender female | |  | |  |
|  | 4. | Transgender male | |  | |  |
|  | 5. | Gender neutral / Non-binary | |  | |  |
|  | 98. | Another gender__________ | |  | |  |
|  | 99. | Prefer not to answer | |  | |  |

| [AssignBirth] | | | |
| --- | --- | --- | --- |
| **- Show to all.** | | |  |
| S3_AssignBirth. | What sex were you assigned at birth? |  |  |
|  | *Select one response.* |  |  |

|  | **DEFAULT ORDER.** | |  | |  | |
| --- | --- | --- | --- | --- | --- | --- |
|  | 1. | Male | |  | |  |
|  | 2. | Female | |  | |  |
|  | 3. | Prefer not to say | |  | |  |

| [YearBorn] | | | |
| --- | --- | --- | --- |
| **- Show to all.** | | |  |
| **- Programmer Comment: Set current year as max.** | | |  |
| S4_YearBorn. | In what year were you born? |  |  |
|  | *Enter a numeric response.* |  |  |

| [DV_Age] | | |
| --- | --- | --- |
| **- Hidden Question. Applies to all.** | |  |
| **- Terminate Immediately if value provided at DV_Age is less than 18.** | |  |
| **- Programmer Comment: Calculate age based on current year.** | |  |
| **- Autocode Logic: Year(Now()) - YearBorn** | |  |
| DV_Age. | Hidden variable to capture age based on YearBorn |  |
|  | *Enter a numeric response.* |  |

| [DV_AgeRange] | | | |
| --- | --- | --- | --- |
| **- Hidden Question. Applies to all.** | | |  |
| DV_AgeRange. | Hidden variable to capture age ranges. |  |  |
|  | *Select one response.* |  |  |

|  | **DEFAULT ORDER.** | |  | |  | |
| --- | --- | --- | --- | --- | --- | --- |
|  | 1. | 18-24 | | **Autocode if value provided at DV_Age is between (inclusive) 18 and 24.** | |  |
|  | 2. | 25-34 | | **Autocode if value provided at DV_Age is between (inclusive) 25 and 34.** | |  |
|  | 3. | 35-44 | | **Autocode if value provided at DV_Age is between (inclusive) 35 and 44.** | |  |
|  | 4. | 45-54 | | **Autocode if value provided at DV_Age is between (inclusive) 45 and 54.** | |  |
|  | 5. | 55-64 | | **Autocode if value provided at DV_Age is between (inclusive) 55 and 64.** | |  |
|  | 6. | 65-70 | | **Autocode if value provided at DV_Age is between (inclusive) 65 and 70.** | |  |
|  | 7. | 71-75 | | **Autocode if value provided at DV_Age is between (inclusive) 71 and 75.** | |  |
|  | 8. | 76+ | | **Autocode if value provided at DV_Age is greater than or equal to 76.** | |  |

| [Income_PreCOV] | | | |
| --- | --- | --- | --- |
| **- Show to all.** | | |  |
| S5_Income_PreCOV. | We know coronavirus and its impact on the economy affected a lot of people’s incomes. Think back before the pandemic. In 2019, what was your total annual household income, before taxes? |  |  |
|  | *Select one response.* |  |  |

|  | **DEFAULT ORDER.** | |  | |  | |
| --- | --- | --- | --- | --- | --- | --- |
|  | 1. | Under $25,000 | |  | |  |
|  | 2. | $25,000 to $34,999 | |  | |  |
|  | 3. | $35,000 to $49,999 | |  | |  |
|  | 4. | $50,000 to $74,999 | |  | |  |
|  | 5. | $75,000 to $99,999 | |  | |  |
|  | 6. | $100,000 to $149,999 | |  | |  |
|  | 7. | $150,000 or more | |  | |  |
|  | 8. | Not willing to say | |  | |  |

| [DV_IncomeNets] | | | |
| --- | --- | --- | --- |
| **- Hidden Question. Applies to all.** | | |  |
| DV_IncomeNets. | Hidden variable to capture netted income for quotas and weighting. |  |  |
|  | *Select one response.* |  |  |

|  | **DEFAULT ORDER.** | |  | |  | |
| --- | --- | --- | --- | --- | --- | --- |
|  | 1. | Under $35,000 | | **Autocode if respondent selected any codes [1-2] at S5_Income_PreCOV.** | |  |
|  | 2. | $35,000 to $49,999 | | **Autocode if respondent selected code [3] at S5_Income_PreCOV.** | |  |
|  | 3. | $50,000 to $74,999 | | **Autocode if respondent selected code [4] at S5_Income_PreCOV.** | |  |
|  | 4. | $75,000 or more | | **Autocode if respondent selected any codes [5-7] at S5_Income_PreCOV.** | |  |
|  | 5. | Refused | | **Autocode if respondent selected code [8] at S5_Income_PreCOV.** | |  |

| [Ethnicity] | | | |
| --- | --- | --- | --- |
| **- Show to all.** | | |  |
| S6_Ethnicity. | Are you of Hispanic or Latin American origin? |  |  |
|  | *Select all that apply.* |  |  |

|  | **DEFAULT ORDER.** | |  | |  | |
| --- | --- | --- | --- | --- | --- | --- |
|  | 1. | Yes, Hispanic | |  | |  |
|  | 2. | Yes, Latin American | |  | |  |
|  | 3. | No | | **EXCLUSIVE.** | |  |

| [Race] | | |
| --- | --- | --- |
| **- Show to all.** | |  |
| S7_Race. | Which of these categories describe you? |  |
|  | *Select all that apply.* |  |

|  | **DEFAULT ORDER.** | |  | |  | |
| --- | --- | --- | --- | --- | --- | --- |
|  | 1. | Asian | |  | |  |
|  | 2. | Black or African American | |  | |  |
|  | 3. | Middle Eastern or North African | |  | |  |
|  | 4. | Native American or Alaska Native | |  | |  |
|  | 5. | Pacific Islander or Native Hawaiian | |  | |  |
|  | 6. | White | |  | |  |
|  | 7. | Only Hispanic or Latin American origin | | **EXCLUSIVE. Show if respondent selected any codes [1-2] at S6_Ethnicity.** | |  |

| [DV_RaceEthnicityNet] | | | |
| --- | --- | --- | --- |
| **- Hidden Question. Applies to all.** | | |  |
| **- Terminate Immediately if respondent did NOT select code [2] at DV_RaceEthnicityNet.** | | |  |
| DV_RaceEthnicityNet. | Hidden variable to capture Ethnicity Net for weighting/quota. |  |  |
|  | *Select one response.* |  |  |

|  | **DEFAULT ORDER.** | |  | |  | |
| --- | --- | --- | --- | --- | --- | --- |
|  | 1. | Hispanic | | **Autocode if respondent selected any codes [1-2] at S6_Ethnicity.** | |  |
|  | 2. | Black or African-American | | **Autocode if respondent selected code [2] at S7_Race AND respondent did NOT select code [1] at DV_RaceEthnicityNet.** | |  |
|  | 3. | Asian or Pacific Islander | | **Autocode if respondent selected any codes [1, 5] at S7_Race AND respondent did NOT select codes [1-2] at DV_RaceEthnicityNet.** | |  |
|  | 4. | Native American/Alaska Native | | **Autocode if respondent selected code [4] at S7_Race AND respondent did NOT select codes [1-3] at DV_RaceEthnicityNet.** | |  |
|  | 5. | Caucasian or White | | **Autocode if respondent selected any codes [3, 6] at S7_Race AND respondent did NOT select codes [1-4] at DV_RaceEthnicityNet.** | |  |

| [Zip] | | |
| --- | --- | --- |
| **- Show to all.** | |  |
| **- Programmer Comment: Allow up to 3 tries. Else, hard term.** | |  |
| S8_Zip. | What is your 5-digit zip code? |  |
|  |  |  |

|  |  |  | **MIN: 5. MAX: 5.** |
| --- | --- | --- | --- |

| [DV_Region] | | | |
| --- | --- | --- | --- |
| **- Hidden Question. Applies to all.** | | |  |
| DV_Region. | Hidden variable to capture Region based on zip |  |  |
|  | *Select one response.* |  |  |

|  | **DEFAULT ORDER.** | |  | |  | |
| --- | --- | --- | --- | --- | --- | --- |
|  | 1. | Midwest | |  | |  |
|  | 2. | Northeast | |  | |  |
|  | 3. | South | |  | |  |
|  | 4. | West | |  | |  |

| [DV_State] | | |
| --- | --- | --- |
| **- Hidden Question. Applies to all.** | |  |
| DV_State. | Automark state based on ZIP |  |
|  |  |  |

|  | **DEFAULT ORDER.** | |  | |  | |
| --- | --- | --- | --- | --- | --- | --- |
|  | 1. | Alabama | |  | |  |
|  | 2. | Alaska | |  | |  |
|  | 3. | Arizona | |  | |  |
|  | 4. | Arkansas | |  | |  |
|  | 5. | California | |  | |  |
|  | 6. | Colorado | |  | |  |
|  | 7. | Connecticut | |  | |  |
|  | 8. | Delaware | |  | |  |
|  | 9. | Florida | |  | |  |
|  | 10. | Georgia | |  | |  |
|  | 11. | Hawaii | |  | |  |
|  | 12. | Idaho | |  | |  |
|  | 13. | Illinois | |  | |  |
|  | 14. | Indiana | |  | |  |
|  | 15. | Iowa | |  | |  |
|  | 16. | Kansas | |  | |  |
|  | 17. | Kentucky | |  | |  |
|  | 18. | Louisiana | |  | |  |
|  | 19. | Maine | |  | |  |
|  | 20. | Maryland | |  | |  |
|  | 21. | Massachusetts | |  | |  |
|  | 22. | Michigan | |  | |  |
|  | 23. | Minnesota | |  | |  |
|  | 24. | Mississippi | |  | |  |
|  | 25. | Missouri | |  | |  |
|  | 26. | Montana | |  | |  |
|  | 27. | Nebraska | |  | |  |
|  | 28. | Nevada | |  | |  |
|  | 29. | New Hampshire | |  | |  |
|  | 30. | New Jersey | |  | |  |
|  | 31. | New Mexico | |  | |  |
|  | 32. | New York | |  | |  |
|  | 33. | North Carolina | |  | |  |
|  | 34. | North Dakota | |  | |  |
|  | 35. | Ohio | |  | |  |
|  | 36. | Oklahoma | |  | |  |
|  | 37. | Oregon | |  | |  |
|  | 38. | Pennsylvania | |  | |  |
|  | 39. | Rhode Island | |  | |  |
|  | 40. | South Carolina | |  | |  |
|  | 41. | South Dakota | |  | |  |
|  | 42. | Tennessee | |  | |  |
|  | 43. | Texas | |  | |  |
|  | 44. | Utah | |  | |  |
|  | 45. | Vermont | |  | |  |
|  | 46. | Virginia | |  | |  |
|  | 47. | Washington | |  | |  |
|  | 48. | Washington D.C. | |  | |  |
|  | 49. | West Virginia | |  | |  |
|  | 50. | Wisconsin | |  | |  |
|  | 51. | Wyoming | |  | |  |

| [SensitiveIndustry] | | | |
| --- | --- | --- | --- |
| **- Show to all.** | | |  |
| **- Terminate Immediately if respondent selected any codes [2-4] at S9_SensitiveIndustry.** | | |  |
| S9_SensitiveIndustry. | Are you currently employed by any of the following? |  |  |
|  | *Select all that apply.* |  |  |

|  | **RANDOMIZE LIST.** | |  | |  | |
| --- | --- | --- | --- | --- | --- | --- |
|  | 1. | A public relations or advertising agency | |  | |  |
|  | 2. | A marketing or marketing research company | | **Terminate immediately.** | |  |
|  | 3. | A company that makes or distributes pharmaceutical products | | **Terminate immediately.** | |  |
|  | 4. | A hospital, medical clinic, doctor's office or any other job in the medical field | | **Terminate immediately.** | |  |
|  | 5. | A furniture company | |  | |  |
|  | 6. | A banking or financial services company | |  | |  |
|  | 99. | None of the above | | **ANCHOR. EXCLUSIVE.** | |  |

| [HealthConditions] | | | |
| --- | --- | --- | --- |
| **- Show to all.** | | |  |
| **- Terminate Immediately if respondent did NOT select code [2] at S10_HealthConditions.** | | |  |
| S10_HealthConditions. | Which of the following health conditions, if any, have you been diagnosed with by a doctor or healthcare provider? |  |  |
|  | *Select all that apply.* |  |  |

|  | **RANDOMIZE LIST.** | |  | |  | |
| --- | --- | --- | --- | --- | --- | --- |
|  | 1. | High cholesterol | |  | |  |
|  | 2. | Breast cancer | |  | |  |
|  | 3. | Lung cancer | |  | |  |
|  | 4. | Melanoma | |  | |  |
|  | 5. | Thyroid cancer | |  | |  |
|  | 6. | High blood pressure | |  | |  |
|  | 7. | Diabetes | |  | |  |
|  | 8. | Obesity | |  | |  |
|  | 9. | Heart disease | |  | |  |
|  | 99. | None of the above | | **ANCHOR. EXCLUSIVE.** | |  |

| [CancerStageNow] | | | |
| --- | --- | --- | --- |
| **- Show to all.** | | |  |
| S11_CancerStageNow. | At what stage is the breast cancer **currently**?  Breast cancer stage is usually assigned as a number on a scale of 1 to 4; "1" describing early cancer that remains within its original location, and "4" describing advanced cancer that has spread outside the breast. |  |  |
|  | *Select one response.* |  |  |

|  | **DEFAULT ORDER.** | |  | |  | |
| --- | --- | --- | --- | --- | --- | --- |
|  | 1. | Stage 1 (Early) – The cancer is small and contained within the organ it started in; it has not spread to the lymph nodes or distant organs | |  | |  |
|  | 2. | Stage 2 – The cancer is growing and MAY have spread to the lymph nodes, but has not spread to distant organs | |  | |  |
|  | 3. | Stage 3 – The cancer is larger, and has spread to the lymph nodes or muscle but has not spread to distant organs | |  | |  |
|  | 4. | Stage 4 (Late/Advanced/Metastatic) – The cancer has spread to distant organs or lymph nodes far from the breast such as bone, liver, brain or lung | |  | |  |
|  | 5. | In remission – I have been told I have no evidence of disease or no longer have cancer | |  | |  |

| [RemissionCancerStage] | | | |
| --- | --- | --- | --- |
| **- Show if respondent selected code [5] at S11_CancerStageNow.** | | |  |
| S12_RemissionCancerStage. | At what stage was the breast cancer right before you went into remission?  Breast cancer stage is usually expressed as a number on a scale of 1 to 4; "1" describing early cancer that remains within its original location, and "4" describing advanced cancer that has spread outside the breast. |  |  |
|  | *Select one response.* |  |  |

|  | **DEFAULT ORDER.** | |  | |  | |
| --- | --- | --- | --- | --- | --- | --- |
|  | 1. | Stage 1 (Early) – The cancer was small and contained within the organ it started in; it had not spread to the lymph nodes or distant organs | |  | |  |
|  | 2. | Stage 2 – The cancer was growing and MAY have spread to the lymph nodes, but had not spread to distant organs | |  | |  |
|  | 3. | Stage 3 – The cancer was larger, and had spread to the lymph nodes or muscle but had not spread to distant organs | |  | |  |
|  | 4. | Stage 4 (Late/Advanced/Metastatic) – The cancer has spread to distant organs or lymph nodes far from the breast such as bone, liver, brain or lung | |  | |  |

| [DV_CancerStageNow] | | | |
| --- | --- | --- | --- |
| **- Hidden Question. Applies to all.** | | |  |
| DV_CancerStageNow. | Hidden variable to capture respondents' breast cancer stage now |  |  |
|  | *Select one response.* |  |  |

|  | **DEFAULT ORDER.** | |  | |  | |
| --- | --- | --- | --- | --- | --- | --- |
|  | 1. | Current Stage I | | **Autocode if respondent selected code [1] at S11_CancerStageNow.** | |  |
|  | 2. | Current Stage II | | **Autocode if respondent selected code [2] at S11_CancerStageNow.** | |  |
|  | 3. | Current Stage III | | **Autocode if respondent selected code [3] at S11_CancerStageNow.** | |  |
|  | 4. | Current Stage IV | | **Autocode if respondent selected code [4] at S11_CancerStageNow.** | |  |
|  | 5. | In Remission Stage I | | **Autocode if respondent selected code [1] at S12_RemissionCancerStage.** | |  |
|  | 6. | In Remission Stage II | | **Autocode if respondent selected code [2] at S12_RemissionCancerStage.** | |  |
|  | 7. | In Remission Stage III | | **Autocode if respondent selected code [3] at S12_RemissionCancerStage.** | |  |
|  | 8. | In Remission Stage IV | | **Autocode if respondent selected code [4] at S12_RemissionCancerStage.** | |  |

| [DV_mBCVseBC] | | | |
| --- | --- | --- | --- |
| **- Hidden Question. Applies to all.** | | |  |
| DV_mBCVseBC. | Hidden variable to capture respondents who are mBC vs. eBC |  |  |
|  | *Select one response.* |  |  |

|  | **DEFAULT ORDER.** | |  | |  | |
| --- | --- | --- | --- | --- | --- | --- |
|  | 1. | mBC | | **Autocode if respondent selected any codes [4, 8] at DV_CancerStageNow.** | |  |
|  | 2. | eBC | | **Autocode if respondent selected any codes [1-3, 5-7] at DV_CancerStageNow.** | |  |

| [MolecSubType] | | | |
| --- | --- | --- | --- |
| **- Show to all.** | | |  |
| S13_MolecSubType. | Which of the following describes your breast cancer? |  |  |
|  | *Select all that apply.* |  |  |

|  | **DEFAULT ORDER.** | |  | |  | |
| --- | --- | --- | --- | --- | --- | --- |
|  | 1. | HR+ / Hormone-receptor positive | | **EXCLUSIVE.** | |  |
|  | 2. | HR- / Hormone-receptor negative | |  | |  |
|  | 3. | Triple-negative | |  | |  |
|  | 97. | Don’t know | | **EXCLUSIVE.** | |  |

| [HRPosMolecSubType] | | | |
| --- | --- | --- | --- |
| **- Show if respondent selected any codes [1, 97] at S13_MolecSubType.** | | |  |
| S14_HRPosMolecSubType. | Which of the following describes your breast cancer? |  |  |
|  | *Select all that apply.* |  |  |

|  | **RANDOMIZE LIST.** | |  | |  | |
| --- | --- | --- | --- | --- | --- | --- |
|  | 1. | ER+ / Estrogen receptor-positive | |  | |  |
|  | 2. | PR+ / Progesterone receptor-positive | |  | |  |
|  | 97. | Don't know | | **ANCHOR. EXCLUSIVE.** | |  |
|  | 99. | None of these | | **ANCHOR. EXCLUSIVE.** | |  |

| [MolecSubDesc] | | | |
| --- | --- | --- | --- |
| **- Show if respondent selected any codes [1-2, 97] at S13_MolecSubType.** | | |  |
| S15_MolecSubDesc. | Which of the following describes your breast cancer? |  |  |
|  | *Select one response.* |  |  |

|  | **DEFAULT ORDER.** | |  | |  | |
| --- | --- | --- | --- | --- | --- | --- |
|  | 1. | HER2+ | |  | |  |
|  | 2. | HER2- | |  | |  |
|  | 97. | Don’t know | | **ANCHOR.** | |  |

| [CancerStageDx] | | | |
| --- | --- | --- | --- |
| **- Show to all.** | | |  |
| S16_CancerStageDx. | At what stage was the breast cancer **when you first received the breast cancer diagnosis**?  Breast cancer stage is usually assigned as a number on a scale of 1 to 4; "1" describing early cancer that remains within its original location, and "4" describing advanced cancer that has spread outside the breast. |  |  |
|  | *Select one response.* |  |  |

|  | **DEFAULT ORDER.** | |  | |  | |
| --- | --- | --- | --- | --- | --- | --- |
|  | 1. | Stage 1 (Early) – The cancer is small and contained within the organ it started in; it has not spread to the lymph nodes or distant organs | |  | |  |
|  | 2. | Stage 2 – The cancer is growing and MAY have spread to the lymph nodes, but has not spread to distant organs | |  | |  |
|  | 3. | Stage 3 – The cancer is larger, and has spread to the lymph nodes or muscle but has not spread to distant organs | |  | |  |
|  | 4. | Stage 4 (Late/Advanced/Metastatic) – The cancer has spread to distant organs or lymph nodes far from the breast such as bone, liver, brain or lung | |  | |  |

| [BCDiagnosisTiming] | | | |
| --- | --- | --- | --- |
| **- Show to all.** | | |  |
| S17_BCDiagnosisTiming. | How long ago were you **first** diagnosed with breast cancer? |  |  |
|  | *Select one response.* |  |  |

|  | **DEFAULT ORDER.** | |  | |  | |
| --- | --- | --- | --- | --- | --- | --- |
|  | 1. | Within the past 6 months | |  | |  |
|  | 2. | 7 - 12 months ago | |  | |  |
|  | 3. | 1 - 5 years ago | |  | |  |
|  | 4. | 6 - 10 years ago | |  | |  |
|  | 5. | 11 - 15 years ago | |  | |  |
|  | 6. | Longer than 15 years ago | |  | |  |

| [BCFamHistory] | | | |
| --- | --- | --- | --- |
| **- Show to all.** | | |  |
| S18_BCFamHistory. | Do you have a family history of breast cancer? |  |  |
|  | *Select one response.* |  |  |

|  | **DEFAULT ORDER.** | |  | |  | |
| --- | --- | --- | --- | --- | --- | --- |
|  | 1. | Yes | |  | |  |
|  | 2. | No | |  | |  |
|  | 3. | I don't know | |  | |  |

| [CancerTreatments] | | | |
| --- | --- | --- | --- |
| **- Show to all.** | | |  |
| S19_CancerTreatments. | What treatment(s) prescribed by a doctor, if any, are you taking for breast cancer? |  |  |
|  | *Select all that apply.* |  |  |

|  | **RANDOMIZE LIST.** | |  | |  | |
| --- | --- | --- | --- | --- | --- | --- |
|  | 1. | Chemotherapy (such as Abraxane, capecitabine, carboplatin, cyclophosphamide, docetaxel, doxorubicin, gemcitabine, Halaven, Ixempra, paclitaxel, or vinorelbine) | |  | |  |
|  | 2. | Hormone therapy (such as anastrozole, exemestane, Faslodex, letrozole, tamoxifen, or Zoladex) | |  | |  |
|  | 3. | Targeted therapy (such as Afinitor, Herceptin, Ibrance, Kadcyla, Kisqali, Lynparza, Nerlynx, Perjeta, Piqray, Talzenna, Tykerb, or Verzenio) | |  | |  |
|  | 4. | Immunotherapy (such as Keytruda, Tecentriq, or Opdivo) | |  | |  |
|  | 5. | Radiation | |  | |  |
|  | 6. | Surgery (within the past 3 months) | |  | |  |
|  | 7. | Treatment to fight my cancer, but I’m not sure what type | |  | |  |
|  | 8. | Supportive or palliative care (to manage symptoms of my breast cancer) | |  | |  |
|  | 98. | Not receiving any treatment, but I receive regular check-ups | | **ANCHOR. EXCLUSIVE.** | |  |
|  | 99. | None of the above | | **ANCHOR. EXCLUSIVE.** | |  |

| [DV_TreatmentExp] | | | |
| --- | --- | --- | --- |
| **- Hidden Question. Applies to all.** | | |  |
| DV_TreatmentExp. | Hidden variable to capture whether respondents have had treatment for breast cancer |  |  |
|  | *Select one response.* |  |  |

|  | **DEFAULT ORDER.** | |  | |  | |
| --- | --- | --- | --- | --- | --- | --- |
|  | 1. | Had treatment | | **Autocode if respondent selected any codes [1-7, 98] at S19_CancerTreatments.** | |  |
|  | 2. | Has never had treatment | | **Autocode if respondent selected code [99] at S19_CancerTreatments.** | |  |

| [LastTreatTiming] | | | |
| --- | --- | --- | --- |
| **- Show if respondent selected code [1] at DV_TreatmentExp.** | | |  |
| S20_LastTreatTiming. | How long ago was the last treatment you had for the breast cancer? |  |  |
|  | *Select all that apply.* |  |  |

|  | **RANDOMIZE LIST.** | |  | |  | |
| --- | --- | --- | --- | --- | --- | --- |
|  | 1. | Within the past 6 months | |  | |  |
|  | 2. | 7 - 12 months ago | |  | |  |
|  | 3. | 1 - 5 years ago | |  | |  |
|  | 4. | 6 - 10 years ago | |  | |  |
|  | 5. | 11 - 15 years ago | |  | |  |
|  | 6. | Longer than 15 years ago | |  | |  |

| [TrialFunnel] | | | |
| --- | --- | --- | --- |
| **- Show to all.** | | |  |
| S21_TrialFunnel. | What is your level of awareness and experience with clinical trials for breast cancer? |  |  |
|  | *Select one response.* |  |  |

|  | **DEFAULT ORDER.** | |  | |  | |
| --- | --- | --- | --- | --- | --- | --- |
|  | 1. | I’ve never heard of clinical trials | |  | |  |
|  | 2. | I’ve heard of clinical trials, but don’t know anything about them | |  | |  |
|  | 3. | I’m familiar with clinical trials, but have never participated in one for breast cancer | |  | |  |
|  | 4. | I have participated in a clinical trial for breast cancer before | |  | |  |
|  | 5. | I am currently in a clinical trial program for breast cancer | |  | |  |

| [DV_TrialExperience] | | | |
| --- | --- | --- | --- |
| **- Hidden Question. Applies to all.** | | |  |
| DV_TrialExperience. | Hidden variable to capture whether respondents have participated in a clinical trial |  |  |
|  | *Select one response.* |  |  |

|  | **DEFAULT ORDER.** | |  | |  | |
| --- | --- | --- | --- | --- | --- | --- |
|  | 1. | Participated | | **Autocode if respondent selected any codes [4-5] at S21_TrialFunnel.** | |  |
|  | 2. | Not Participated | | **Autocode if respondent did NOT select codes [4-5] at S21_TrialFunnel.** | |  |

| [AdvocateBC] | | | |
| --- | --- | --- | --- |
| **- Show to all.** | | |  |
| S22_AdvocateBC. | Do you advocate, raise awareness, or do outreach regularly for breast cancer, in the breast cancer community?  If so, through what channels? |  |  |
|  | *Select all that apply.* |  |  |

|  | **RANDOMIZE LIST.** | | | |  | | | |  | | | |  |
| --- | --- | --- | --- | --- | --- | --- | --- | --- | --- | --- | --- | --- | --- |
|  | 1. | | Advocating on social media | | | |  | | | |  |  |  |
|  | 2. | | Sharing my story in writing | | | |  | | | |  |  |  |
|  | 3. | | Speaking at panels or events | | | |  | | | |  |  |  |
|  | 4. | | Organizing or moderating forums | | | |  | | | |  |  |  |
|  | | **DEFAULT ORDER.** | | | |  | | | |  | | | |
|  | | 5. | | Leading or running an advocacy organization | | | |  | | | |  |  |
|  | | 6. | | Leading or running a support group | | | |  | | | |  |  |
|  | | 7. | | Leading or running another initiative / project | | | |  | | | |  |  |
|  | 98. | | Other channels / programs__________ | | | | **ANCHOR.** | | | |  |  |  |
|  | 99. | | I don't personally do any of the above | | | | **ANCHOR. EXCLUSIVE.** | | | |  |  |  |

| [OfferSupportBC] | | | |
| --- | --- | --- | --- |
| **- Show to all.** | | |  |
| S23_OfferSupportBC. | How frequently, if at all, do you personally offer advice and emotional support to other breast cancer patients? |  |  |
|  | *Select one response.* |  |  |

|  | **DEFAULT ORDER.** | |  | |  | |
| --- | --- | --- | --- | --- | --- | --- |
|  | 1. | A couple times a week | |  | |  |
|  | 2. | Once a week | |  | |  |
|  | 3. | Once a month | |  | |  |
|  | 4. | Once every few months | |  | |  |
|  | 5. | Once or twice a year, or less often | |  | |  |
|  | 99. | I don't personally offer advice and emotional support to other breast cancer patients | |  | |  |

| [DV_BCChoir] | | | |
| --- | --- | --- | --- |
| **- Hidden Question. Applies to all.** | | |  |
| DV_BCChoir. | Hidden variable to capture highly involved patients in breast cancer community "choir" vs. not |  |  |
|  | *Select one response.* |  |  |

|  | **DEFAULT ORDER.** | |  | |  | |
| --- | --- | --- | --- | --- | --- | --- |
|  | 1. | Highly involved in choir | | **Autocode if respondent selected any codes [1-7, 98] at S22_AdvocateBC AND respondent selected any codes [1-4] at S23_OfferSupportBC.** | |  |
|  | 2. | Not highly involved in choir | | **Autocode if respondent did NOT select code [1] at DV_BCChoir.** | |  |

| **Screener terminations** |
| --- |

| **Question number** | **Termination logic statement** | **Termination type** |
| --- | --- | --- |
| DV_Age | Terminate Immediately if value provided at DV_Age is less than 18 | Immediately |
| DV_RaceEthnicityNet | Terminate Immediately if respondent did NOT select code [2] at DV_RaceEthnicityNet | Immediately |
| S9_SensitiveIndustry | Terminate Immediately if respondent selected any codes [2-4] at S9_SensitiveIndustry | Immediately |
| S10_HealthConditions | Terminate Immediately if respondent did NOT select code [2] at S10_HealthConditions | Immediately |

### Clinical Trials Perceptions - Among Aware/Participated

| [ClinicalTrials_Intro] | | | |
| --- | --- | --- | --- |
| **- Show if respondent selected any codes [2-5] at S21_TrialFunnel.** | | |  |
| DT_ClinicalTrials_Intro. | Thank you for your interest in our survey! We appreciate your time and effort. Your input is very important to us.  In the questions that follow, we would like to ask you about clinical trials. |  |  |
|  |  |  |  |

| [TrialAwareSource] | | | |
| --- | --- | --- | --- |
| **- Show if respondent selected any codes [2-5] at S21_TrialFunnel.** | | |  |
| A1_TrialAwareSource. | Earlier you mentioned that you are aware of clinical trials for breast cancer. How did you **first** hear / find out about clinical trials for breast cancer? |  |  |
|  | *Select one response.* |  |  |

|  | **RANDOMIZE LIST.** | | | |  | | | |  | | | |  |
| --- | --- | --- | --- | --- | --- | --- | --- | --- | --- | --- | --- | --- | --- |
|  | | **RANDOMIZE WITHIN.** | | | |  | | | |  | | | |
|  | | 1. | | Saw a poster or pamphlet for it in a doctor's office | | | |  | | | |  |  |
|  | | 2. | | A doctor or healthcare provider mentioned it | | | |  | | | |  |  |
|  | 3. | | Heard about it on TV | | | |  | | | |  |  |  |
|  | | **DEFAULT ORDER.** | | | |  | | | |  | | | |
|  | | 4. | | Saw an ad pop up online | | | |  | | | |  |  |
|  | | 5. | | Saw videos online | | | |  | | | |  |  |
|  | | 6. | | Through my own online research via search engines, such as Google or Yahoo (**not** social media) | | | |  | | | |  |  |
|  | | 7. | | Through a post on my social media feed (e.g., Facebook, Instagram, Twitter) | | | |  | | | |  |  |
|  | | 8. | | Through a breast cancer group or community on social media (e.g., Facebook) | | | |  | | | |  |  |
|  | | **RANDOMIZE WITHIN.** | | | |  | | | |  | | | |
|  | | 9. | | From another breast cancer patient | | | |  | | | |  |  |
|  | | 10. | | From a breast cancer support group | | | |  | | | |  |  |
|  | | 11. | | From a breast cancer conference | | | |  | | | |  |  |
|  | | 12. | | From a friend or family member | | | |  | | | |  |  |
|  | | 13. | | From a celebrity or influencer with personal or family experience with breast cancer | | | |  | | | |  |  |
|  | | **RANDOMIZE WITHIN.** | | | |  | | | |  | | | |
|  | | 14. | | An educational program at a hospital | | | |  | | | |  |  |
|  | | 15. | | An educational program in a school setting | | | |  | | | |  |  |
|  | 16. | | A clergy member or faith-based community | | | |  | | | |  |  |  |
|  | 98. | | Other__________ | | | | **ANCHOR.** | | | |  |  |  |

| [AllSourcesAware] | | | |
| --- | --- | --- | --- |
| **- Show if respondent selected any codes [2-5] at S21_TrialFunnel. Only show row stubs not selected at A1_TrialAwareSource.** | | |  |
| A2_AllSourcesAware. | What are **all the other ways** that you have learned about clinical trials for breast cancer? |  |  |
|  | *Select all that apply.* |  |  |

|  | **RANDOMIZE LIST.** | | | |  | | | |  | | | |  |
| --- | --- | --- | --- | --- | --- | --- | --- | --- | --- | --- | --- | --- | --- |
|  | | **RANDOMIZE WITHIN.** | | | |  | | | |  | | | |
|  | | 1. | | Saw a poster or pamphlet for it in a doctor's office | | | |  | | | |  |  |
|  | | 2. | | A doctor or healthcare provider mentioned it | | | |  | | | |  |  |
|  | 3. | | Heard about it on TV | | | |  | | | |  |  |  |
|  | | **DEFAULT ORDER.** | | | |  | | | |  | | | |
|  | | 4. | | Saw an ad pop up online | | | |  | | | |  |  |
|  | | 5. | | Saw videos online | | | |  | | | |  |  |
|  | | 6. | | Through my own online research via search engines, such as Google or Yahoo (**not** social media) | | | |  | | | |  |  |
|  | | 7. | | Through a post on my social media feed (e.g., Facebook, Instagram, Twitter) | | | |  | | | |  |  |
|  | | 8. | | Through a breast cancer group or community on social media (e.g., Facebook) | | | |  | | | |  |  |
|  | | **RANDOMIZE WITHIN.** | | | |  | | | |  | | | |
|  | | 9. | | From another breast cancer patient | | | |  | | | |  |  |
|  | | 10. | | From a breast cancer support group | | | |  | | | |  |  |
|  | | 11. | | From a breast cancer conference | | | |  | | | |  |  |
|  | | 12. | | From a friend or family member | | | |  | | | |  |  |
|  | | 13. | | From a celebrity or influencer with personal or family experience with breast cancer | | | |  | | | |  |  |
|  | | **RANDOMIZE WITHIN.** | | | |  | | | |  | | | |
|  | | 14. | | An educational program at a hospital | | | |  | | | |  |  |
|  | | 15. | | An educational program in a school setting | | | |  | | | |  |  |
|  | 16. | | A clergy member or faith-based community | | | |  | | | |  |  |  |
|  | 98. | | Other__________ | | | | **ANCHOR.** | | | |  |  |  |
|  | 99. | | None of the above | | | | **ANCHOR. EXCLUSIVE.** | | | |  |  |  |

| [TrialPerceptions] | | | |
| --- | --- | --- | --- |
| **- Show if respondent selected any codes [2-5] at S21_TrialFunnel.** | | |  |
| A3_TrialPerceptions. | How much do you agree or disagree with the following statements regarding clinical trials as a treatment for breast cancer?  *Clinical trials...* |  |  |
|  | *Select one response for each.* |  |  |

| Rows 4-5: RANDOMIZE WITHIN. |
| --- |

| **COLS: DEFAULT ORDER.**  **ROWS: RANDOMIZE LIST.** | **Disagree strongly** | **Disagree somewhat** | **Neither agree nor disagree** | **Agree somewhat** | **Agree strongly** |  |  |
| --- | --- | --- | --- | --- | --- | --- | --- |
| 1. Are life-saving | *1* | *2* | *3* | *4* | *5* |  | |
| 2. Bring a sense of security | *1* | *2* | *3* | *4* | *5* |  | |
| 3. Allow for health stability | *1* | *2* | *3* | *4* | *5* |  | |
| 4. Can cause serious side effects | *1* | *2* | *3* | *4* | *5* |  | |
| 5. Can cause long-term side effects | *1* | *2* | *3* | *4* | *5* |  | |
| 6. Are dangerous | *1* | *2* | *3* | *4* | *5* |  | |
| 7. Might not give you a real treatment | *1* | *2* | *3* | *4* | *5* |  | |
| 8. Involve experimenting on patients | *1* | *2* | *3* | *4* | *5* |  | |
| 9. Are not genuine care | *1* | *2* | *3* | *4* | *5* |  | |
|  |  |  |  |  |  |  | |

| [TrialExpectations] | | | |
| --- | --- | --- | --- |
| **- Show if respondent selected any codes [2-5] at S21_TrialFunnel.** | | |  |
| A4_TrialExpectations. | How much do you agree or disagree that the following are reasonable expected outcomes from participating in a clinical trial for breast cancer? |  |  |
|  | *Select one response for each.* |  |  |

| **COLS: DEFAULT ORDER.**  **ROWS: RANDOMIZE LIST.** | **Disagree strongly** | **Disagree somewhat** | **Neither agree nor disagree** | **Agree somewhat** | **Agree strongly** |  |  |
| --- | --- | --- | --- | --- | --- | --- | --- |
| 1. Receive potential life-saving treatment | *1* | *2* | *3* | *4* | *5* |  | |
| 2. Receive the newest treatments I might not otherwise have access to | *1* | *2* | *3* | *4* | *5* |  | |
| 3. Receive better patient care | *1* | *2* | *3* | *4* | *5* |  | |
| 4. Get financial assistance for treatments I might not be able to afford otherwise | *1* | *2* | *3* | *4* | *5* |  | |
| 5. Help find treatments to benefit others like me in the future | *1* | *2* | *3* | *4* | *5* |  | |
| 6. Risk potential harm from unproven treatments | *1* | *2* | *3* | *4* | *5* |  | |
| 7. Have more frequent doctor appointments | *1* | *2* | *3* | *4* | *5* |  | |
| 8. Have to treat my condition for a longer period of time | *1* | *2* | *3* | *4* | *5* |  | |
|  |  |  |  |  |  |  | |

| [TrialDiscuss] | | | |
| --- | --- | --- | --- |
| **- Show if respondent selected any codes [2-3] at S21_TrialFunnel.** | | |  |
| A5_TrialDiscuss. | Have you ever talked to your doctor about the possibility of participating in a clinical trial for breast cancer? |  |  |
|  | *Select one response.* |  |  |

|  | **DEFAULT ORDER.** | |  | |  | |
| --- | --- | --- | --- | --- | --- | --- |
|  | 1. | Yes | |  | |  |
|  | 2. | No | |  | |  |

| [DV_DiscussedTrialsDr] | | | |
| --- | --- | --- | --- |
| **- Hidden Question. Applies to all.** | | |  |
| DV_DiscussedTrialsDr. | Hidden variable to capture whether respondents have discussed clinical trials with doctor. Respondents who have participated are autocoded as having discussed. |  |  |
|  | *Select one response.* |  |  |

|  | **DEFAULT ORDER.** | |  | |  | |
| --- | --- | --- | --- | --- | --- | --- |
|  | 1. | Yes | | **Autocode if respondent selected code [1] at DV_TrialExperience OR respondent selected code [1] at A5_TrialDiscuss.** | |  |
|  | 2. | No | | **Autocode if respondent selected code [2] at DV_TrialExperience AND respondent selected code [2] at A5_TrialDiscuss.** | |  |

###

#### Clinical Trial Conversations - Among Who Discussed with Health Care Provider

| [TrialDiscussTimes] | | | |
| --- | --- | --- | --- |
| **- Show if respondent selected code [1] at DV_DiscussedTrialsDr.** | | |  |
| A6_TrialDiscussTimes. | How many times have you talked to your doctor about possibly participating in a clinical trial for breast cancer? |  |  |
|  | *Select one response.* |  |  |

|  | **DEFAULT ORDER.** | |  | |  | |
| --- | --- | --- | --- | --- | --- | --- |
|  | 1. | Once | |  | |  |
|  | 2. | Twice | |  | |  |
|  | 3. | Three or four times | |  | |  |
|  | 4. | More than 4 times | |  | |  |

| [TrialDiscussInitial] | | | |
| --- | --- | --- | --- |
| **- Show if respondent selected code [1] at DV_DiscussedTrialsDr.** | | |  |
| A7_TrialDiscussInitial. | During the **first** time you talked to your doctor about possibly participating in a clinical trial for breast cancer, who brought it up? |  |  |
|  | *Select one response.* |  |  |

|  | **DEFAULT ORDER.** | |  | |  | |
| --- | --- | --- | --- | --- | --- | --- |
|  | 1. | I brought it up to my doctor | |  | |  |
|  | 2. | Someone who accompanied me to my appointment brought it up to my doctor | |  | |  |
|  | 3. | My doctor brought it up to me | |  | |  |

| [TrialEmotionDiscuss] | | | |
| --- | --- | --- | --- |
| **- Show if respondent selected code [1] at DV_DiscussedTrialsDr.** | | |  |
| A8_TrialEmotionDiscuss. | And which of the following best describes your emotions during the **first** time you talked to your doctor about possibly participating in a clinical trial for breast cancer? |  |  |
|  | *Select one response.* |  |  |

|  | **RANDOMIZE LIST.** | |  | |  | |
| --- | --- | --- | --- | --- | --- | --- |
|  | 1. | Excited | |  | |  |
|  | 2. | Inspired | |  | |  |
|  | 3. | Satisfied | |  | |  |
|  | 4. | Pleased | |  | |  |
|  | 5. | Secure | |  | |  |
|  | 6. | At ease | |  | |  |
|  | 7. | Nervous | |  | |  |
|  | 8. | Angry | |  | |  |
|  | 9. | Troubled | |  | |  |
|  | 10. | Unhappy | |  | |  |
|  | 11. | Bored | |  | |  |
|  | 12. | Down | |  | |  |

| [TrialDiscussTopics] | | | |
| --- | --- | --- | --- |
| **- Show if respondent selected code [1] at DV_DiscussedTrialsDr.** | | |  |
| A9_TrialDiscussTopics. | When you talked to your doctor about possibly participating in a clinical trial for breast cancer, which of the following topics did you talk about? |  |  |
|  | *Select all that apply.* |  |  |

|  | **RANDOMIZE LIST.** | |  | |  | |
| --- | --- | --- | --- | --- | --- | --- |
|  | 1. | What type of treatment the clinical trial is for | |  | |  |
|  | 2. | How the treatment would work for me personally | |  | |  |
|  | 3. | Any evidence showing it could work for me | |  | |  |
|  | 4. | How effective the treatment is | |  | |  |
|  | 5. | How the clinical trial treatment compares with other existing treatment options | |  | |  |
|  | 6. | What the potential risks and benefits are for my health | |  | |  |
|  | 7. | What the potential side effects are | |  | |  |
|  | 8. | For how long I would be participating in the clinical trial | |  | |  |
|  | 9. | What would happen if I stopped participating in the trial | |  | |  |
|  | 10. | If and how my confidentiality would be protected | |  | |  |
|  | 11. | How much it costs/if it is covered by insurance | |  | |  |
|  | 12. | How frequently I would need to see a doctor during treatment | |  | |  |
|  | 13. | Where the clinical trial locations are | |  | |  |
|  | 14. | How I would get to/from the clinical trial location | |  | |  |
|  | 15. | How to handle my (and my family's) daily needs while I am participating in the clinical trial | |  | |  |
|  | 98. | Other__________ | | **ANCHOR.** | |  |
|  | 99. | None of the above | | **ANCHOR. EXCLUSIVE.** | |  |

| [TrialTopicUnderstand] | | | |
| --- | --- | --- | --- |
| **- Show if respondent selected code [1] at DV_DiscussedTrialsDr AND respondent did NOT select code [99] at A9_TrialDiscussTopics. Only show row stubs selected at A9_TrialDiscussTopics.** | | |  |
| A10_TrialTopicUnderstand. | And how well did you feel that you understood each of the following topics after talking to your doctor? |  |  |
|  | *Select one response for each.* |  |  |

| **COLS: DEFAULT ORDER.**  **ROWS: RANDOMIZE LIST.** | **Did not understand at all** | **Did not understand most of it** | **Understood somewhat** | **Understood well** | **Understood extremely well** |  |  |
| --- | --- | --- | --- | --- | --- | --- | --- |
| 1. What type of treatment the clinical trial is for | *1* | *2* | *3* | *4* | *5* |  | |
| 2. How the treatment would work for me personally | *1* | *2* | *3* | *4* | *5* |  | |
| 3. Any evidence showing it could work for me | *1* | *2* | *3* | *4* | *5* |  | |
| 4. How effective the treatment is | *1* | *2* | *3* | *4* | *5* |  | |
| 5. How the clinical trial treatment compares with other existing treatment options | *1* | *2* | *3* | *4* | *5* |  | |
| 6. What the potential risks and benefits are for my health | *1* | *2* | *3* | *4* | *5* |  | |
| 7. What the potential side effects are | *1* | *2* | *3* | *4* | *5* |  | |
| 8. For how long I would be participating in the clinical trial | *1* | *2* | *3* | *4* | *5* |  | |
| 9. What would happen if I stopped participating in the trial | *1* | *2* | *3* | *4* | *5* |  | |
| 10. If and how my confidentiality would be protected | *1* | *2* | *3* | *4* | *5* |  | |
| 11. How much it costs/if it is covered by insurance | *1* | *2* | *3* | *4* | *5* |  | |
| 12. How frequently I would need to see a doctor during treatment | *1* | *2* | *3* | *4* | *5* |  | |
| 13. Where the clinical trial locations are | *1* | *2* | *3* | *4* | *5* |  | |
| 14. How I would get to/from the clinical trial location | *1* | *2* | *3* | *4* | *5* |  | |
| 15. How to handle my (and my family's) daily needs while I am participating in the clinical trial | *1* | *2* | *3* | *4* | *5* |  | |
|  |  |  |  |  |  |  | |

| [TrialTopicsMoreInfo] | | | |
| --- | --- | --- | --- |
| **- Show if respondent selected code [1] at DV_DiscussedTrialsDr.** | | |  |
| A11_TrialTopicsMoreInfo. | Which of the following topics would you have wanted more information about? |  |  |
|  | *Select all that apply.* |  |  |

|  | **RANDOMIZE LIST.** | |  | |  | |
| --- | --- | --- | --- | --- | --- | --- |
|  | 1. | What type of treatment the clinical trial is for | |  | |  |
|  | 2. | How the treatment would work for me personally | |  | |  |
|  | 3. | Any evidence showing it could work for me | |  | |  |
|  | 4. | How effective the treatment is | |  | |  |
|  | 5. | How the clinical trial treatment compares with other existing treatment options | |  | |  |
|  | 6. | What the potential risks and benefits are for my health | |  | |  |
|  | 7. | What the potential side effects are | |  | |  |
|  | 8. | For how long I would be participating in the clinical trial | |  | |  |
|  | 9. | What would happen if I stopped participating in the trial | |  | |  |
|  | 10. | If and how my confidentiality would be protected | |  | |  |
|  | 11. | How much it costs/if it is covered by insurance | |  | |  |
|  | 12. | How frequently I would need to see a doctor during treatment | |  | |  |
|  | 13. | Where the clinical trial locations are | |  | |  |
|  | 14. | How I would get to/from the clinical trial location | |  | |  |
|  | 15. | How to handle my (and my family's) daily needs while I am participating in the clinical trial | |  | |  |
|  | 98. | Other__________ | | **ANCHOR.** | |  |
|  | 99. | None of the above | | **ANCHOR. EXCLUSIVE.** | |  |

| [TrialInformedRating] | | | |
| --- | --- | --- | --- |
| **- Show if respondent selected code [1] at DV_DiscussedTrialsDr.** | | |  |
| A12_TrialInformedRating. | Overall, after all of the conversations you had with your doctor, how informed did you feel about the clinical trial? |  |  |
|  | *Select one response.* |  |  |

|  | **DEFAULT ORDER.** | |  | |  | |
| --- | --- | --- | --- | --- | --- | --- |
|  | 1. | Not at all informed | |  | |  |
|  | 2. | Not informed | |  | |  |
|  | 3. | Somewhat informed | |  | |  |
|  | 4. | Very informed | |  | |  |
|  | 5. | Extremely informed | |  | |  |

| [TrialEligibility] | | | |
| --- | --- | --- | --- |
| **- Show if respondent selected code [1] at A5_TrialDiscuss.** | | |  |
| A13_TrialEligibility. | When you talked to your doctor about possibly participating in a clinical trial for breast cancer, which of the following best describes your case? |  |  |
|  | *Select one response.* |  |  |

|  | **DEFAULT ORDER.** | |  | |  | |
| --- | --- | --- | --- | --- | --- | --- |
|  | 1. | I was eligible and selected, but decided not to participate | |  | |  |
|  | 2. | I was eligible, but not selected | |  | |  |
|  | 3. | I was not eligible | |  | |  |
|  | 97. | I don't know | |  | |  |

| [ReasonsNotParticipat] | | | |
| --- | --- | --- | --- |
| **- Show if respondent selected code [1] at A13_TrialEligibility.** | | |  |
| A14_ReasonsNotParticipat. | And how much did each of the following items influence your decision not to participate, after talking about it with your doctor? |  |  |
|  | *Select one response for each.* |  |  |

| **COLS: DEFAULT ORDER.**  **ROWS: RANDOMIZE LIST.** | **No influence at all** | **Not much influence** | **Some influence** | **A great deal of influence** | **Was a deciding factor in my decision** |  |  |
| --- | --- | --- | --- | --- | --- | --- | --- |
| 1. Not being in control of my treatment process | *1* | *2* | *3* | *4* | *5* |  | |
| 2. Not knowing what treatment I'm receiving | *1* | *2* | *3* | *4* | *5* |  | |
| 3. The doctor not adequately addressing the potential drawbacks of the clinical trial | *1* | *2* | *3* | *4* | *5* |  | |
| 4. I preferred my current treatment to the trial treatment offered | *1* | *2* | *3* | *4* | *5* |  | |
| 5. I did not completely understand how the clinical trial treatment would help me | *1* | *2* | *3* | *4* | *5* |  | |
| 6. I felt like it would cost me additional expenses to participate | *1* | *2* | *3* | *4* | *5* |  | |
| 7. I had too many concerns about my ability to carry on my daily life | *1* | *2* | *3* | *4* | *5* |  | |
| 8. It would put too much strain on my family | *1* | *2* | *3* | *4* | *5* |  | |
| 9. I did not feel I was at a point of needing to take an experimental treatment | *1* | *2* | *3* | *4* | *5* |  | |
| 10. Possibility of making my private medical information public | *1* | *2* | *3* | *4* | *5* |  | |
| 11. I felt rushed or pressured to make a decision | *1* | *2* | *3* | *4* | *5* |  | |
| 12. I did not have an established enough relationship to trust the person who introduced the trial to me | *1* | *2* | *3* | *4* | *5* |  | |
|  |  |  |  |  |  |  | |

| [MorethanDiag] | | | |
| --- | --- | --- | --- |
| **- Show to all.** | | |  |
| A22_MorethanDiag. | Have you received more than one breast cancer diagnosis? |  |  |
|  | *Select one response.* |  |  |

|  | **DEFAULT ORDER.** | |  | |  | |
| --- | --- | --- | --- | --- | --- | --- |
|  | 1. | Yes | |  | |  |
|  | 2. | No | |  | |  |

| [AdditionalDiag] | | | |
| --- | --- | --- | --- |
| **- Show if respondent selected code [1] at A22_MorethanDiag.** | | |  |
| A23_AdditionalDiag. | Was the additional diagnosis a recurrence of the initial breast cancer or a new breast cancer diagnosis? |  |  |
|  | *Select all that apply.* |  |  |

|  | **DEFAULT ORDER.** | |  | |  | |
| --- | --- | --- | --- | --- | --- | --- |
|  | 1. | It was a recurrence of the initial breast cancer | |  | |  |
|  | 2. | It was a new breast cancer | |  | |  |
|  | 3. | I don't know | | **EXCLUSIVE.** | |  |

#### Clinical Trial Barriers and Motivators - Among ALL

| [TrialEmotionNow] | | | |
| --- | --- | --- | --- |
| **- Show to all.** | | |  |
| A15_TrialEmotionNow. | "Clinical trials are research studies designed to evaluate whether an investigational drug is safe and effective for use in humans. Participants are given specific investigational treatments and researchers closely monitor the results to help determine if the drug should be approved for wider use. The U.S. Food and Drug Administration (FDA) has strict rules that govern how clinical studies are conducted, and requires that an investigational drug be proven safe and effective before it can be widely used in the United States."  After reading this description about clinical trials, which of the following best describes your emotions? |  |  |
|  | *Select one response.* |  |  |

|  | **RANDOMIZE LIST.** | |  | |  | |
| --- | --- | --- | --- | --- | --- | --- |
|  | 1. | Excited | |  | |  |
|  | 2. | Inspired | |  | |  |
|  | 3. | Satisfied | |  | |  |
|  | 4. | Pleased | |  | |  |
|  | 5. | Secure | |  | |  |
|  | 6. | At ease | |  | |  |
|  | 7. | Nervous | |  | |  |
|  | 8. | Angry | |  | |  |
|  | 9. | Troubled | |  | |  |
|  | 10. | Unhappy | |  | |  |
|  | 11. | Bored | |  | |  |
|  | 12. | Down | |  | |  |

| [TrialLogisticBarrier] | | | |
| --- | --- | --- | --- |
| **- Show to all.** | | |  |
| A16_TrialLogisticBarrier. | If you wanted to participate in a clinical trial for breast cancer and were selected as a participant, which of the following, if any, do you think would limit your ability to participate? |  |  |
|  | *Select all that apply.* |  |  |

|  | **RANDOMIZE LIST.** | |  | |  | |
| --- | --- | --- | --- | --- | --- | --- |
|  | 1. | Not having easy access to transportation | |  | |  |
|  | 2. | Extra financial expenses not covered by the trial | |  | |  |
|  | 3. | Not having access to support services, such as counselling for mental health | |  | |  |
|  | 4. | It would interfere with my commitments at work | |  | |  |
|  | 5. | Not having sufficient health insurance | |  | |  |
|  | 6. | Living far away from healthcare facilities | |  | |  |
|  | 7. | Not being able to get childcare | |  | |  |
|  | 98. | Other__________ | | **ANCHOR.** | |  |
|  | 99. | None of the above | | **ANCHOR. EXCLUSIVE.** | |  |

| [TrialEmotionBarrier] | | | |
| --- | --- | --- | --- |
| **- Show to all.** | | |  |
| A17_TrialEmotionBarrier. | Which of the following, if any, are concerns you have about participating in clinical trials for breast cancer? |  |  |
|  | *Select all that apply.* |  |  |

|  | **RANDOMIZE LIST.** | |  | |  | |
| --- | --- | --- | --- | --- | --- | --- |
|  | 1. | Receiving an experimental treatment instead of an approved treatment | |  | |  |
|  | 2. | Not having control over my treatment process | |  | |  |
|  | 3. | My doctor gets financial benefits for getting clinical trial participants, whether it's the best option for me or not | |  | |  |
|  | 4. | The trial does not guarantee the best health outcome for me | |  | |  |
|  | 5. | It could make my condition worse | |  | |  |
|  | 6. | I'm skeptical of clinical trials due to historical experiences in my community | |  | |  |
|  | 7. | I don't trust the healthcare system to make decisions about my health due to past personal negative experiences | |  | |  |
|  | 8. | Side effects that haven't previously been discovered | |  | |  |
|  | 9. | Not knowing the duration of the treatment | |  | |  |
|  | 10. | I may get a placebo | |  | |  |
|  | 11. | I may get a sugar pill | |  | |  |
|  | 12. | It would go against my personal beliefs and/or faith | |  | |  |
|  | 13. | It would go against family's beliefs and/or faith | |  | |  |
|  | 14. | The possibility of making my private medical information public | |  | |  |
|  | 15. | My family would be worried for me | |  | |  |
|  | 98. | Other__________ | | **ANCHOR.** | |  |
|  | 99. | None of the above | | **ANCHOR. EXCLUSIVE.** | |  |

| [TrialMotivators] | | | |
| --- | --- | --- | --- |
| **- Show to all.** | | |  |
| A18_TrialMotivators. | Below are some potential outcomes from participating in clinical trials for breast cancer. How much do each of the following encourage you to participate in one? |  |  |
|  | *Select one response for each.* |  |  |

| **COLS: DEFAULT ORDER.**  **ROWS: RANDOMIZE LIST.** | **Does not encourage me at all** | **Does not encourage me much** | **Encourages me somewhat** | **Encourages me a lot** | **Encourages me extremely** |  |  |
| --- | --- | --- | --- | --- | --- | --- | --- |
| 1. I may get a new treatment for my condition before it is available to everyone else | *1* | *2* | *3* | *4* | *5* |  | |
| 2. I would play a more active role in my own health care | *1* | *2* | *3* | *4* | *5* |  | |
| 4. I may help find treatments to benefit others like me in the future | *1* | *2* | *3* | *4* | *5* |  | |
| 5. My treatment may be more effective than the standard approach | *1* | *2* | *3* | *4* | *5* |  | |
| 6. I will get more frequent health evaluations as part of the clinical trial | *1* | *2* | *3* | *4* | *5* |  | |
| 7. I will get more comprehensive health evaluations as part of the clinical trial | *1* | *2* | *3* | *4* | *5* |  | |
| 8. I may receive monetary compensation in addition to my treatment expenses | *1* | *2* | *3* | *4* | *5* |  | |
| 9. My treatment expenses would be fully covered | *1* | *2* | *3* | *4* | *5* |  | |
|  |  |  |  |  |  |  | |

| [TrialLogisticAssist] | | | |
| --- | --- | --- | --- |
| **- Show to all.** | | |  |
| A19_TrialLogisticAssist. | How much, if at all, would any of the following **make it easier** for you to participate in a clinical trial for breast cancer? |  |  |
|  | *Select one response for each.* |  |  |

| **COLS: DEFAULT ORDER.**  **ROWS: RANDOMIZE LIST.** | **Would not help at all** | **Would not help much** | **Would help somewhat** | **Would help very much** | **Would be required for me to participate in a clinical trial** |  |  |
| --- | --- | --- | --- | --- | --- | --- | --- |
| 1. Financial assistance for my medical care | *1* | *2* | *3* | *4* | *5* |  | |
| 2. Access to support services, such as counselling for mental health | *1* | *2* | *3* | *4* | *5* |  | |
| 3. Transportation to/from the clinical trial site | *1* | *2* | *3* | *4* | *5* |  | |
| 4. Childcare while I am participating in the clinical trial | *1* | *2* | *3* | *4* | *5* |  | |
| 5. Household assistance (meals, cleaning, daily care) while I am participating in the clinical trial | *1* | *2* | *3* | *4* | *5* |  | |
| 6. Having study visits conducted in my home or place of work | *1* | *2* | *3* | *4* | *5* |  | |
|  |  |  |  |  |  |  | |

| [SourcesTrust] | | | |
| --- | --- | --- | --- |
| **- Show to all.** | | |  |
| A20_SourcesTrust. | When it comes to receiving accurate and reliable information about clinical trials for breast cancer, how much do you **trust** each of the following sources? |  |  |
|  | *Select one response for each.* |  |  |

| Rows 1-2: RANDOMIZE WITHIN. |
| --- |
| Rows 4-8: DEFAULT ORDER. |
| Rows 9-13: RANDOMIZE WITHIN. |
| Rows 14-15: RANDOMIZE WITHIN. |

| **COLS: DEFAULT ORDER.**  **ROWS: RANDOMIZE LIST.** | **I do not trust it at all** | **I do not have much trust in it** | **I have some trust in it** | **I have a high level of trust in it** | **I have an extremely high level of trust in it** |  |  |
| --- | --- | --- | --- | --- | --- | --- | --- |
| 1. A poster or pamphlet in a doctor's office | *1* | *2* | *3* | *4* | *5* |  | |
| 2. A healthcare provider | *1* | *2* | *3* | *4* | *5* |  | |
| 3. TV content | *1* | *2* | *3* | *4* | *5* |  | |
| 4. Online ads | *1* | *2* | *3* | *4* | *5* |  | |
| 5. Online videos | *1* | *2* | *3* | *4* | *5* |  | |
| 6. Online search engines, such as Google or Yahoo (**not** social media) | *1* | *2* | *3* | *4* | *5* |  | |
| 7. Social media posts (e.g., Facebook, Instagram, Twitter) | *1* | *2* | *3* | *4* | *5* |  | |
| 8. Social media groups or communities (e.g., Facebook) | *1* | *2* | *3* | *4* | *5* |  | |
| 9. Another breast cancer patient | *1* | *2* | *3* | *4* | *5* |  | |
| 10. A breast cancer support group | *1* | *2* | *3* | *4* | *5* |  | |
| 11. A breast cancer conference | *1* | *2* | *3* | *4* | *5* |  | |
| 12. Friends and family members | *1* | *2* | *3* | *4* | *5* |  | |
| 13. A celebrity or influencer with personal or family experience with breast cancer | *1* | *2* | *3* | *4* | *5* |  | |
| 14. An educational program at a hospital | *1* | *2* | *3* | *4* | *5* |  | |
| 15. An educational program in a school setting | *1* | *2* | *3* | *4* | *5* |  | |
| 16. A clergy member or faith-based community | *1* | *2* | *3* | *4* | *5* |  | |
|  |  |  |  |  |  |  | |

| [CommunitySourcesPref] | | | |
| --- | --- | --- | --- |
| **- Show to all.** | | |  |
| A21_CommunitySourcesPref. | Through which of the following community-based touchpoints/networks would you be open to receiving information about clinical trials for breast cancer? |  |  |
|  | *Select all that apply.* |  |  |

|  | **RANDOMIZE LIST.** | |  | |  | |
| --- | --- | --- | --- | --- | --- | --- |
|  | 1. | Greek life chapters | |  | |  |
|  | 2. | Church / other places of worship | |  | |  |
|  | 3. | Beauty salons and barbershops | |  | |  |
|  | 4. | College campuses | |  | |  |
|  | 98. | Other__________ | | **ANCHOR.** | |  |
|  | 99. | None of the above | | **ANCHOR. EXCLUSIVE.** | |  |

### Relationship with Health Care Providers - Among All

| [HCPs_Intro] | | | |
| --- | --- | --- | --- |
| **- Show to all.** | | |  |
| DT_HCPs_Intro. | Thank you for your answers so far!  In the questions that follow, we would like to better understand your relationship with the doctor **primarily responsible for managing your breast cancer treatment.** |  |  |
|  |  |  |  |

| [ComfortHCP] | | | |
| --- | --- | --- | --- |
| **- Show to all.** | | |  |
| B1_ComfortHCP. | How comfortable are you asking your doctor questions about the availability of, or starting, specific treatments for breast cancer? |  |  |
|  | *Select one response.* |  |  |

|  | **DEFAULT ORDER.** | |  | |  | |
| --- | --- | --- | --- | --- | --- | --- |
|  | 1. | Not at all comfortable | |  | |  |
|  | 2. | Not comfortable | |  | |  |
|  | 3. | Somewhat comfortable | |  | |  |
|  | 4. | Comfortable | |  | |  |
|  | 5. | Extremely comfortable | |  | |  |

| [TrustHCP] | | | |
| --- | --- | --- | --- |
| **- Show to all.** | | |  |
| B2_TrustHCP. | How much do you trust your doctor to recommend the best treatments for you for breast cancer? |  |  |
|  | *Select one response.* |  |  |

|  | **DEFAULT ORDER.** | |  | |  | |
| --- | --- | --- | --- | --- | --- | --- |
|  | 1. | I do **not** trust him/her **at all** | |  | |  |
|  | 2. | I do **not** have **much** trust in him/her | |  | |  |
|  | 3. | I have **some** trust in him/her | |  | |  |
|  | 4. | I have a **high** level of trust in him/her | |  | |  |
|  | 5. | I have an **extremely high** level of trust in him/her | |  | |  |

| [SatisfactionHCP] | | | |
| --- | --- | --- | --- |
| **- Show to all.** | | |  |
| B3_SatisfactionHCP. | How satisfied are you with the overall care you receive from your doctor for breast cancer? |  |  |
|  | *Select one response.* |  |  |

|  | **DEFAULT ORDER.** | |  | |  | |
| --- | --- | --- | --- | --- | --- | --- |
|  | 1. | Not at all satisfied | |  | |  |
|  | 2. | Not satisfied | |  | |  |
|  | 3. | Somewhat satisfied | |  | |  |
|  | 4. | Satisfied | |  | |  |
|  | 5. | Extremely satisfied | |  | |  |

| [ViewsHCP] | | | |
| --- | --- | --- | --- |
| **- Show to all.** | | |  |
| B4_ViewsHCP. | How much do you agree or disagree with the following statements about your doctor for breast cancer?  *My doctor...* |  |  |
|  | *Select one response for each.* |  |  |

| **COLS: DEFAULT ORDER.**  **ROWS: RANDOMIZE LIST.** | **Disagree strongly** | **Disagree somewhat** | **Neither agree nor disagree** | **Agree somewhat** | **Agree strongly** |  |  |
| --- | --- | --- | --- | --- | --- | --- | --- |
| 1. Does everything they can do to manage my breast cancer | *1* | *2* | *3* | *4* | *5* |  | |
| 2. Is up-to-date on all the new treatment options | *1* | *2* | *3* | *4* | *5* |  | |
| 3. Is empathetic and easy to talk to | *1* | *2* | *3* | *4* | *5* |  | |
| 4. Takes the time to get to know me as a person | *1* | *2* | *3* | *4* | *5* |  | |
| 5. Takes the time to explain my condition and treatment options until I fully understand | *1* | *2* | *3* | *4* | *5* |  | |
| 6. Has offered me the best available treatment options | *1* | *2* | *3* | *4* | *5* |  | |
| 7. Has helped me access the financial assistance I needed for my treatments | *1* | *2* | *3* | *4* | *5* |  | |
|  |  |  |  |  |  |  | |

### Racial Impact - Among All

| [RaceImpact] | | | |
| --- | --- | --- | --- |
| **- Show to all.** | | |  |
| C1_RaceImpact. | What impact do you think your race has on the **quality** of the medical care treatment you receive for breast cancer? |  |  |
|  | *Select one response.* |  |  |

|  | **DEFAULT ORDER.** | |  | |  | |
| --- | --- | --- | --- | --- | --- | --- |
|  | 1. | Negative impact | |  | |  |
|  | 2. | No impact | |  | |  |
|  | 3. | Positive impact | |  | |  |

| [EquityHealthCare] | | | |
| --- | --- | --- | --- |
| **- Show to all.** | | |  |
| C2_EquityHealthCare. | Which of the following, if any, do you feel would most create a more equitable healthcare experience for you? |  |  |
|  | *Select all that apply.* |  |  |

|  | **RANDOMIZE LIST.** | |  | |  | |
| --- | --- | --- | --- | --- | --- | --- |
|  | 1. | Increased non-White representation in my healthcare team | |  | |  |
|  | 2. | Increased female representation in my healthcare team | |  | |  |
|  | 3. | Opportunity to talk to other women with a race, background and diagnosis like mine | |  | |  |
|  | 4. | Assurance that my doctor has experience treating other breast cancer patients with a race and background like mine | |  | |  |
|  | 5. | More education around breast cancer taught in schools or educational institutions | |  | |  |
|  | 6. | More reliable insurance coverage for my breast cancer needs | |  | |  |
|  | 98. | Other__________ | | **ANCHOR.** | |  |
|  | 99. | None of the above | | **ANCHOR. EXCLUSIVE.** | |  |

| [DrGender] | | | |
| --- | --- | --- | --- |
| **- Show to all.** | | |  |
| C3_DrGender. | What gender do you think your doctor for breast cancer is? |  |  |
|  | *Select one response.* |  |  |

|  | **DEFAULT ORDER.** | |  | |  | |
| --- | --- | --- | --- | --- | --- | --- |
|  | 1. | Male | |  | |  |
|  | 2. | Female | |  | |  |
|  | 3. | Transgender female | |  | |  |
|  | 4. | Transgender male | |  | |  |
|  | 5. | Gender neutral / Non-binary | |  | |  |
|  | 6. | Another gender__________ | | **ANCHOR.** | |  |

| [DrEthnicity] | | | |
| --- | --- | --- | --- |
| **- Show to all.** | | |  |
| C4_DrEthnicity. | What race / ethnicity do you think your doctor for breast cancer is? |  |  |
|  | *Select one response.* |  |  |

|  | **RANDOMIZE LIST.** | |  | |  | |
| --- | --- | --- | --- | --- | --- | --- |
|  | 1. | Hispanic / Latinx | |  | |  |
|  | 2. | Asian or Pacific Islander | |  | |  |
|  | 3. | Black or African-American | |  | |  |
|  | 4. | Middle Eastern or North African | |  | |  |
|  | 5. | Native people of Latin or North America | |  | |  |
|  | 6. | White | |  | |  |

| [DrAgeRange] | | | |
| --- | --- | --- | --- |
| **- Show to all.** | | |  |
| C5_DrAgeRange. | What age range do you think your doctor for breast cancer is? |  |  |
|  | *Select one response.* |  |  |

|  | **DEFAULT ORDER.** | |  | |  | |
| --- | --- | --- | --- | --- | --- | --- |
|  | 1. | Under 30 years old | |  | |  |
|  | 2. | 30-49 years old | |  | |  |
|  | 3. | 50-69 years old | |  | |  |
|  | 4. | 70+ years old | |  | |  |

### Demographics

| [CommInvolvement] | | | |
| --- | --- | --- | --- |
| **- Show to all.** | | |  |
| D1_CommInvolvement. | Since the breast cancer diagnosis, how involved, if at all, have you been with any of the following? |  |  |
|  | *Select one response for each.* |  |  |

| **COLS: DEFAULT ORDER.**  **ROWS: DEFAULT ORDER.** | **Very involved (organized events for this group)** | **Involved (helped out with events others organized)** | **fSomewhat involved (participated actively in events)** | **A little involved (attended some events)** | **Not at all involved** |  |  |
| --- | --- | --- | --- | --- | --- | --- | --- |
| 1. Online support groups or communities on websites or social media | *1* | *2* | *3* | *4* | *5* |  | |
| 2. Local, in-person support groups (or video support groups) | *1* | *2* | *3* | *4* | *5* |  | |
| 3. A breast cancer organization specific to Black women or women of color (e.g., For the Breast of Us, Sisters Network, The Black Breast Cancer Alliance, TOUCH) | *1* | *2* | *3* | *4* | *5* |  | |
| 4. A general breast cancer advocacy organization (e.g., Breastcancer.org, Living Beyond Breast Cancer, Susan G. Komen Foundation, Triple Negative Breast Cancer Foundation, Young Survival Coalition) | *1* | *2* | *3* | *4* | *5* |  | |
| 5. A general cancer organization (e.g., American Cancer Society, Cancer Support Community, LiveStrong) | *1* | *2* | *3* | *4* | *5* |  | |
| 6. Susan Love Foundation | *1* | *2* | *3* | *4* | *5* |  | |
|  |  |  |  |  |  |  | |

| [DV_CommInvolvement] | | | |
| --- | --- | --- | --- |
| **- Hidden Question. Applies to all.** | | |  |
| DV_CommInvolvement. | Hidden variable to capture respondents' level of involvement in community |  |  |
|  | *Select one response.* |  |  |

|  | **DEFAULT ORDER.** | |  | |  | |
| --- | --- | --- | --- | --- | --- | --- |
|  | 1. | Active | | **Autocode if, for at least 2 row(s) at D1_CommInvolvement, the respondent selected any codes [1-3]. PC: Autopunch if at least 2 rows at S16_CommunityInvolvement includes codes 3-5; NOT counting row 1 (Online support groups on websites or social media)** | |  |
|  | 2. | Not Active | | **Autocode if respondent did NOT select code [1] at DV_CommInvolvement.** | |  |

| [Parent] | | | |
| --- | --- | --- | --- |
| **- Show to all.** | | |  |
| D2_Parent. | Are you the parent of any children under 18 years old, who live in your household? |  |  |
|  | *Select one response.* |  |  |

|  | **DEFAULT ORDER.** | |  | |  | |
| --- | --- | --- | --- | --- | --- | --- |
|  | 1. | Yes | |  | |  |
|  | 2. | No | |  | |  |

| [Caretaker] | | | |
| --- | --- | --- | --- |
| **- Show to all.** | | |  |
| D3_Caretaker. | Do you currently have one or more primary caretakers, who help you in emotional and practical ways when it comes to breast cancer treatment? |  |  |
|  | *Select one response.* |  |  |

|  | **DEFAULT ORDER.** | |  | |  | |
| --- | --- | --- | --- | --- | --- | --- |
|  | 1. | Yes | |  | |  |
|  | 2. | No | |  | |  |

| [CaretakersWho] | | | |
| --- | --- | --- | --- |
| **- Show if respondent selected code [1] at D3_Caretaker.** | | |  |
| D4_CaretakersWho. | Which of the following would you describe as primary caretakers for you while you are undergoing cancer treatments? |  |  |
|  | *Select all that apply.* |  |  |

|  | **RANDOMIZE LIST.** | |  | |  | |
| --- | --- | --- | --- | --- | --- | --- |
|  | 1. | Parent(s) | |  | |  |
|  | 2. | A spouse or partner | |  | |  |
|  | 3. | Adult child(ren) | |  | |  |
|  | 4. | Friend(s) | |  | |  |
|  | 5. | Extended family (aunts, uncles, cousins, etc.) | |  | |  |
|  | 99. | Faith-based community | |  | |  |
|  | 98. | Someone else | | **ANCHOR.** | |  |

| [Insurance] | | | |
| --- | --- | --- | --- |
| **- Show to all.** | | |  |
| D5_Insurance. | What type of health insurance, if any, do you currently have? |  |  |
|  | *Select one response.* |  |  |

|  | **DEFAULT ORDER.** | |  | |  | |
| --- | --- | --- | --- | --- | --- | --- |
|  | 1. | Commercial insurance (e.g. from an employer, private insurance) | |  | |  |
|  | 2. | A health plan purchased through the market exchange under the Affordable Care Act | |  | |  |
|  | 3. | Medicare | |  | |  |
|  | 4. | Medicaid | |  | |  |
|  | 5. | Veteran’s or active military healthcare, such as Tricare | |  | |  |
|  | 98. | Another form of health insurance__________ | |  | |  |
|  | 99. | No health insurance/pay out-of-pocket | |  | |  |

| [DrHealthcareFacility] | | | |
| --- | --- | --- | --- |
| **- Show to all.** | | |  |
| D6_DrHealthcareFacility. | What type of facility does the doctor that primarily manages the treatment of the breast cancer work out of? |  |  |
|  | *Select one response.* |  |  |

|  | **DEFAULT ORDER.** | |  | |  | |
| --- | --- | --- | --- | --- | --- | --- |
|  | 1. | Teaching hospital | |  | |  |
|  | 2. | Community hospital (General) | |  | |  |
|  | 3. | Long-Term Care Hospital | |  | |  |
|  | 4. | Center of excellence (specialized program within hospital) | |  | |  |
|  | 5. | Acute Care | |  | |  |
|  | 6. | Private practice | |  | |  |
|  | 98. | Other__________ | | **ANCHOR.** | |  |
|  | 97. | I don't know | | **ANCHOR.** | |  |

| [Education] | | | |
| --- | --- | --- | --- |
| **- Show to all.** | | |  |
| D7_Education. | What is the highest level of education you completed? |  |  |
|  | *Select one response.* |  |  |

|  | **DEFAULT ORDER.** | |  | |  | |
| --- | --- | --- | --- | --- | --- | --- |
|  | 1. | Some high school | |  | |  |
|  | 2. | High school graduate | |  | |  |
|  | 3. | Vocational/trade school | |  | |  |
|  | 4. | Some college | |  | |  |
|  | 5. | College graduate | |  | |  |
|  | 6. | Post-graduate degree | |  | |  |
|  | 7. | Prefer not to say | |  | |  |

| [MaritalStatus] | | | |
| --- | --- | --- | --- |
| **- Show to all.** | | |  |
| D8_MaritalStatus. | What is your marital status? |  |  |
|  | *Select one response.* |  |  |

|  | **DEFAULT ORDER.** | |  | |  | |
| --- | --- | --- | --- | --- | --- | --- |
|  | 1. | Married or living as married | |  | |  |
|  | 2. | Widowed | |  | |  |
|  | 3. | Separated or divorced | |  | |  |
|  | 4. | Single, never been married | |  | |  |

| [Employment] | | | |
| --- | --- | --- | --- |
| **- Show to all.** | | |  |
| D9_Employment. | What is your current employment status? |  |  |
|  | *Select one response.* |  |  |

|  | **DEFAULT ORDER.** | |  | |  | |
| --- | --- | --- | --- | --- | --- | --- |
|  | 1. | Employed full-time | |  | |  |
|  | 2. | Employed part-time | |  | |  |
|  | 3. | Not employed | |  | |  |
|  | 4. | A Homemaker | |  | |  |
|  | 5. | Retired | |  | |  |
|  | 6. | On short-term or long-term disability | |  | |  |
|  | 7. | Currently seeking work | |  | |  |

| [Urbanicity] | | | |
| --- | --- | --- | --- |
| **- Show to all.** | | |  |
| D10_Urbanicity. | What type of  area do you live in? |  |  |
|  | *Select one response.* |  |  |

|  | **DEFAULT ORDER.** | |  | |  | |
| --- | --- | --- | --- | --- | --- | --- |
|  | 1. | An urban setting | |  | |  |
|  | 2. | A suburban setting | |  | |  |
|  | 3. | A smaller city or larger town | |  | |  |
|  | 4. | Countryside or rural setting | |  | |  |

| [DV_UrbanicityABCD] | | | |
| --- | --- | --- | --- |
| **- Hidden Question. Applies to all.** | | |  |
| **- Programmer Comment: Please code based on this database P:\006_Corriveau\Zip Code Databases (US Canada)\DMA\County Size.xlsx** | | |  |
| DV_UrbanicityABCD. | Respondent urbanicity, based on zip code. |  |  |
|  |  |  |  |

|  | **DEFAULT ORDER.** | |  | |  | |
| --- | --- | --- | --- | --- | --- | --- |
|  | 1. | A, part of 1 of the 25 largest metropolitan areas in the US | |  | |  |
|  | 2. | B, 150,000 + population or a part of a metropolitan area | |  | |  |
|  | 3. | C, population size between 40,000 - 150, 000 people | |  | |  |
|  | 4. | D, population size <40,000 people | |  | |  |

# Supplemental Figures


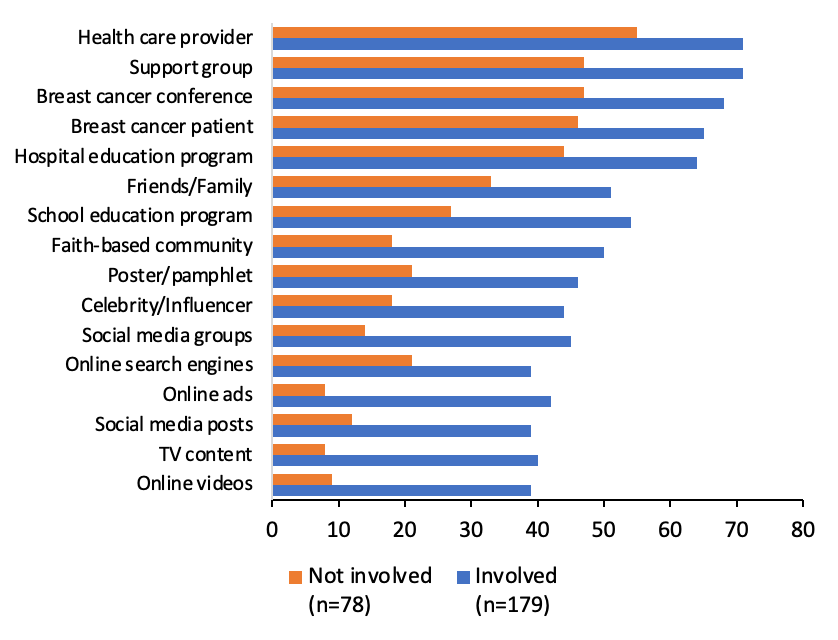


## Supplemental Fig 1

Participants were asked, “When it comes to receiving accurate and reliable information about clinical trials for breast cancer, how much do you trust each of the following sources?” (Question A20, Supplemental Methods). Participants were stratified according to whether they were active in the breast cancer community. For each source, participants who were involved were significantly more likely to indicate that they had a high or extremely high level of trust in the source compared to participants who were not involved in the breast cancer community (*p* < 0.05 in all comparisons).


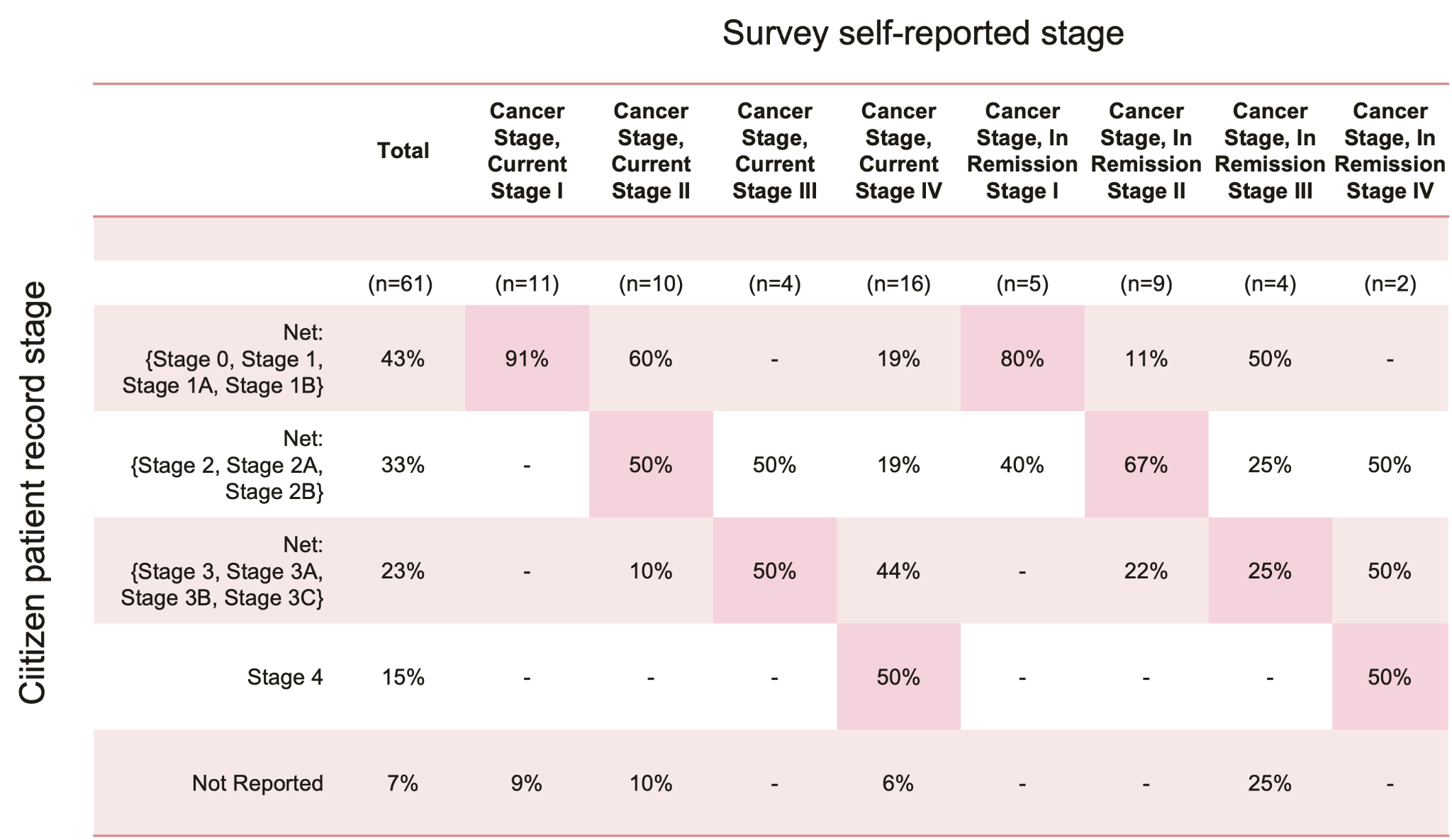


## Supplemental Fig 2

Concordance of breast cancer stage (patient records vs self-reported in quantitative survey) of patients enrolled in Ciitizen who participated in the quantitative survey
